# Material–Medium Performance Transferability of Two Modified Cementitious Grout Systems

**DOI:** 10.3390/ma19214545

**Published:** 2026-10-28

**Authors:** Qizhi Chen, Zhipeng Shu, Hongruyi Wei, Zhongen Qin, Baoping Zou, Shuyi Kong

**Affiliations:** 1 School of Civil Engineering and Architecture, Zhejiang University of Science and Technology, No. 318 Liuhe Road, Xihu District, Hangzhou 310023, China; chenqizhi@zust.edu.cn (Q.C.); 222502859020@zust.edu.cn (Z.S.);; 2 Zhejiang-Singapore Joint Laboratory for Urban Renewal and Future City, Zhejiang University of Science and Technology, Hangzhou 310023, China; 3 Taizhou Traffic Engineering Test Center Co., Ltd., No. 168 Chunchao Road, Jiaojiang District, Taizhou 318001, China

**Keywords:** cementitious grout, material–medium performance transferability, material-scale screening, microstructure, water-to-binder ratio, stress retention, grout body continuity

## Abstract

**Highlights:**

- Material screening favored nominal *W*/*B* = 0.6 in both cementitious grout systems.
- Confined-medium rankings were preserved for GFP but reordered for SS-EA.
- Stable stress responses and grout body continuity supported medium-specific selection.
- Cementitious material selection benefits from validation in the intended host medium.

**Abstract:**

The performance of cementitious grouts in a host medium depends on whether advantages identified by material-scale screening remain effective after placement. This study examined material–medium performance transferability in two fly-ash cementitious grouts modified with glass fiber powder (GFP) or sodium silicate and an expansive agent (SS-EA). Eighteen mixes were screened using bleeding ratio, hardened volume ratio, and compressive and flexural strengths at 1, 3, and 7 d. With each candidate’s additive combination fixed, nominal water-to-binder ratios (W/B) of 0.6, 0.7, and 0.8 were assessed in independently prepared confined-sand specimens. Confinement and staged volume–time control were held constant. Mix preferences were evaluated within each grout system under its formulation convention. Both systems selected nominal W/B=0.6 at the material scale. GFP retained this ranking, with relatively high and balanced stable stress responses in both directions. The preferred SS-EA condition shifted to nominal W/B=0.7, with more favorable stress retention and grout body continuity. These outcomes show that balanced fresh-state and hardened properties do not necessarily preserve mix preference in the material–medium system. Material screening followed by medium-specific validation supports selection that connects cementitious material properties with spatial continuity and short-term post-injection stress retention.

## 1. Introduction

Cementitious grout selection generally begins with the material itself, considering fresh-state stability, rheological and setting behavior, and hardened strength [1,2]. These properties govern whether a grout can be prepared consistently and develop a load-bearing skeleton but cannot fully describe its performance in granular media. Once injected into sand, grout must travel and spread through restricted pores, form a spatially continuous grout body, and establish mechanical support under confinement. Performance therefore becomes a property of the material–medium system [3,4]. This transition is particularly relevant to compensation or corrective grouting, where spatially controlled support is required [5,6].

Material-scale selection involves trade-offs among several properties. Changing the water-to-binder ratio affects stability, setting, and hardened load-bearing capacity simultaneously [7,8]. Fly ash and sodium silicate further modify viscous behavior, strength development, and hardened structure [9,10]. Consequently, the strongest mix may not provide the best balance among bleeding, volume retention, construction-related properties, and strength [11]. Mollamahmutoglu and Avci provided a direct example for microfine Portland cement grout: increasing the water-to-cement ratio improved groutability in medium to fine sand but reduced the unconfined compressive strength of the grouted sand [12]. Transport-related and hardened load-bearing properties can therefore favor different mixes. A material-scale candidate must reconcile competing criteria rather than maximize a single property.

Relative mix performance in granular media also depends on the medium and injection conditions. The water-to-binder ratio, sand gradation, and density influence penetration, spatial distribution, and reinforcement response [3,4,13,14]. Grout particle–pore throat compatibility, viscosity, filtration, and injection conditions jointly govern groutability and penetration distance [15,16,17]. Recent experiments in differently graded coral sands showed that the groutability ranking of cement slurries with three solid-phase particle sizes changed with medium permeability [18]. Relative grout performance thus reflects the combined effects of material properties, medium structure, and injection boundaries.

Previous work has linked grout viscosity, setting, and stability with penetration and the strength or permeability of grouted sand [12,15,16,17,18]. GFP research has also examined the mechanical response of grouted coal-gangue aggregates [19], while GFP and SS-EA material studies provide the formulation context [19,20]. The question examined here is whether a candidate selected by multiple material properties retains its preferred nominal W/B when its additive combination is fixed and assessed by short-term bidirectional stress development and retention in confined sand. This sequential comparison adds an injection-stage stress criterion to the material-property and groutability evidence.

Here, we examine cementitious material–medium performance transferability by testing whether balanced material properties preserve mix preference after placement in a controlled host medium. We first screened 18 GFP and SS-EA mixes separately within each system. With each selected additive combination fixed, nominal water-to-binder ratios (W/B) of 0.6, 0.7, and 0.8 were compared under the system-specific formulation conventions. Independently prepared medium-dense sand specimens provided a common validation setting with fixed lateral confinement and three-stage volume–time-controlled injection. Stage I stress development and retention in both directions, together with recovered grout body continuity, were used to reassess within-system rankings. The objective was to connect fresh-state stability and hardened load-bearing properties with medium-dependent spatial continuity and stress retention during the recorded holding period, identifying ranking preservation or reordering.

## 2. Materials and Methods

### 2.1. Experimental Concept and Two-Stage Comparison

**Figure 1:**
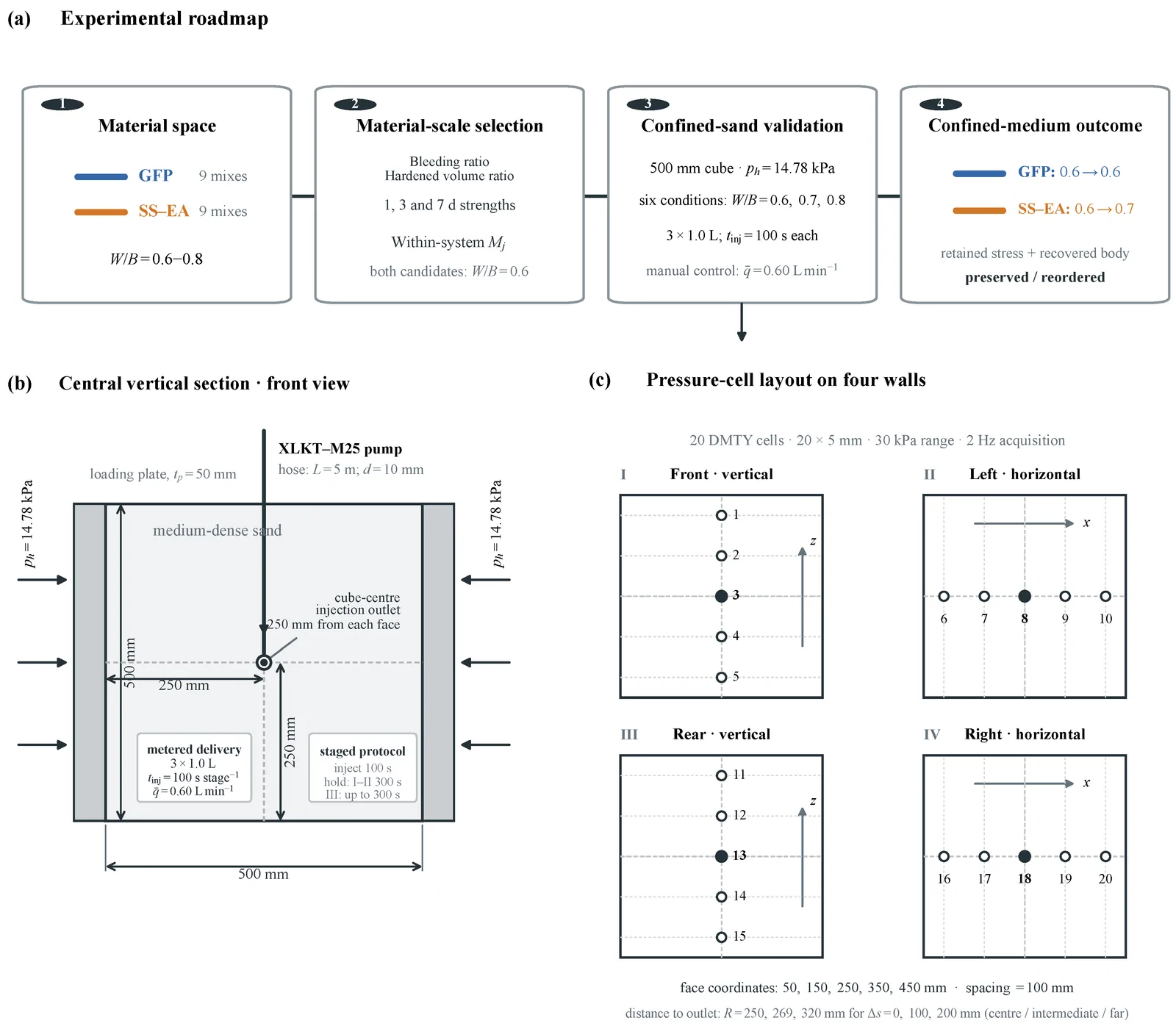
Material-scale screening and confined-sand validation. GFP and SS-EA denote glass-fiber-powder-modified and sodium-silicate–UEA-modified fly-ash cementitious grouts, respectively. (**a**) Two-stage workflow covering the mix design space, candidate screening using fresh-state and strength properties, confined direct grouting, and identification of ranking preservation or ranking reordering. Blue and orange denote GFP and SS-EA, respectively. (**b**) Central vertical section of the 500 mm × 500 mm sand box viewed from the front. A 50 mm-thick loading plate applies lateral confinement of $p_{h}=14.78$ kPa. The outlet of the 10 mm-diameter injection pipe is at the box center. Each condition receives three 1.0 L injections, each lasting 100 s, with a manually controlled stage-averaged volumetric flow rate of 0.60 L $\min^{-1}$. (**c**) Sensor positions on four monitoring faces. Vertical lines 1#–5# and 11#–15# are on the front and rear faces; horizontal lines 6#–10# and 16#–20# are on the left and right faces. The five positions on each face are 50, 150, 250, 350, and 450 mm from the boundary, at 100 mm intervals. Central, intermediate, and outer positions are approximately 250, 269, and 320 mm from the outlet.

The experimental design comprised sequential material-scale screening and confined-sand validation (Figure 1). The first stage examined W/B and additive combinations separately for GFP and SS-EA. Material-scale candidates were identified using bleeding ratio, hardened volume ratio, and compressive and flexural strengths at 1, 3, and 7 d. SEM, XRD, and FTIR were used to assess whether the candidates’ hardened structures were consistent with their material-property rankings.

The second stage fixed each candidate’s additive combination and compared nominal W/B=0.6, 0.7, and 0.8 under that system’s formulation convention. Ranking preservation denotes the material candidate’s nominal W/B remaining preferred in this fixed-additive sequence; a change in preferred W/B denotes ranking reordering. This reassessment compares W/B preference within each fixed-additive sequence. Stage I central vertical and horizontal stable increments and retention ratios determined the response comparison. Final recovered morphology supplied complementary spatial evidence after the full staged protocol. Later stages characterized cumulative responses within each test. Cross-system analysis compared these within-system outcomes.

### 2.2. Raw Materials and Mix Design

#### 2.2.1. Raw Materials

The materials were ordinary Portland cement (P·O 42.5; OPC), Class II pulverized fly ash (PFA), alkali-free glass fiber powder (GFP), UEA expansive agent, and liquid sodium silicate (SS). OPC was supplied by Fushun Cement Co., Ltd. (Fushun, China), with a density of 3.18 g ${\mathrm{cm}}^{-3}$ and a specific surface area of 358 $m^{2}$ ${\mathrm{kg}}^{-1}$. PFA, supplied by Shaanxi Zhengyuan Fly Ash Comprehensive Utilization Co., Ltd. (Xixian New Area, China), had an 8.2% residue on a 45 μm sieve and a water requirement of 93.02%. GFP was supplied by Fengfengshun Stone Powder Factory (Shijiazhuang, China), with particle sizes of 10–350 μm and filament diameters of 11–17 μm. UEA was supplied by Laiyang Hongxiang Construction Admixture Factory (Laiyang, China). SS was supplied by Jiashan Yourui Refractory Materials Co., Ltd. (Jiashan, Jiaxing, China), with a density of 1.37 g ${\mathrm{cm}}^{-3}$, a Baumé degree of 38.5 °Bé, a modulus of 3.3, a solids content of 35.5%, and a pH of 10–13. OPC and PFA met GB 175 [21] and GB/T 1596 [22], respectively.

Table 1 summarizes the available chemical indices; oxide coverage is partial for PFA and UEA.

#### 2.2.2. Mix Design

GFP grout contained OPC, PFA, and GFP; SS-EA grout contained OPC, PFA, UEA, and SS. Each system comprised nine material-scale mixes at nominal W/B=0.6, 0.7, and 0.8 under its original formulation convention (Table 2). GFP mixes varied the PFA and GFP contents, both expressed as percentages of the combined OPC and PFA mass. SS-EA mixes varied the PFA, UEA, and SS contents, all expressed as percentages of OPC mass.

The nominal formulation conventions were retained. For GFP, *W* is separately added water and $B=m_{\mathrm{OPC}}+m_{\mathrm{PFA}}$; GFP is an external addition. For SS-EA, *W* is separately added water and $B=m_{\mathrm{OPC}}$; PFA, UEA, and the SS solution are external additions on this basis. To express both systems on a common mass-accounting basis, the calculated ratio is $w/s_{\mathrm{calc}}=\left(W+0.645m_{\mathrm{SS}}\right)/\left(m_{\mathrm{OPC}}+m_{\mathrm{PFA}}+m_{\mathrm{GFP}}+m_{\mathrm{UEA}}+0.355m_{\mathrm{SS}}\right)$, where $m_{\mathrm{SS}}$ is solution mass and unused components are zero. This calculation treats powders as dry and uses the reported SS solids fraction of 0.355. It is a formulation-based ratio, not a measured free-water ratio. Table 2 and Table 3 retain the nominal labels and add $w/s_{\mathrm{calc}}$; Supplementary Table S3 gives component masses and water contributions. Within each fixed-additive injection sequence, the conversion increases monotonically with nominal W/B, preserving the order of the observed responses. Equal nominal W/B values do not establish compositional equivalence across systems.

### 2.3. Material-Scale Screening

#### 2.3.1. Specimen Preparation

All components were weighed according to the designed mix proportions. OPC, PFA, and either GFP or UEA were dry-mixed for 2–3 min. For GFP grout, the measured water was added, followed by slow mixing for 3–5 min and then rapid mixing. For SS-EA grout, SS was first mixed with the measured water and added to the dry materials, followed by slow mixing for 3–5 min and then rapid mixing. Strength specimens were cast in 40 mm × 40 mm × 160 mm molds and consolidated by vibration. They were demolded after 24 h and cured at 20±1 °C and at least 90% relative humidity. Microstructural samples were taken from the corresponding hardened specimens to compare the final structural organization of different formulations.

#### 2.3.2. Fresh-State Stability and Mechanical Properties

Bleeding ratio and hardened volume ratio were measured by allowing grout to stand in a 500 mL graduated cylinder. After 1 h, the separated free-water volume was read every 15 min until two consecutive readings were stable. The hardened grout volume was read at 24 h. The bleeding ratio $B_{r}$ and hardened volume ratio *S* were calculated using Equations (1) and (2):

(1) $$B_{r}=\frac{V_{w}}{V_{0}}\times 100\%,$$

(2) $$S=\frac{V_{s}}{V_{0}}\times 100\%,$$

where $V_{w}$ is the final separated free-water volume, $V_{s}$ is the hardened grout volume at 24 h, and $V_{0}$ is the initial grout volume.

Specimen dimensions and compressive and flexural loading rates followed GB/T 17671 [23]; the loading rates were 2400±200 N s^−1^ and 50±10 N s^−1^, respectively. Three prisms from the same preparation batch were tested for each mix–age combination. Compressive strength was determined using one selected half-prism from each parent prism. Strengths are reported as arithmetic mean ± sample SD across the three specimens (n=3); SD describes within-batch specimen variability.

#### 2.3.3. Candidate Screening Using a Noncompensatory Score

Because grout selection must constrain both fresh-state stability and hardened load-bearing capacity, the bleeding resistance, hardened volume ratio, and age-specific compressive and flexural strengths were combined in a noncompensatory screening metric. Min–max normalization was performed separately for GFP and SS-EA. The hardened volume ratio and both strengths used the positive form in Equation (3); bleeding ratio used the inverse form in Equation (4):

(3) $$N_{+}=\frac{x-x_{\min}}{x_{\max}-x_{\min}},$$

(4) $$N_{-}=\frac{x_{\max}-x}{x_{\max}-x_{\min}}.$$

Each mix had eight normalized indicators. The noncompensatory balance score for mix *j* was defined by Equation (5):

(5) $$M_{j}=\min_{k}\left(N_{j,k}\right).$$

The lowest-performing indicator controls $M_{j}$. A high value for one property therefore cannot offset a marked weakness in bleeding resistance, hardened volume ratio, or strength. The mix with the largest $M_{j}$ in each tested design space was selected as that system’s material-scale candidate. Rule sensitivity was assessed by omitting each indicator in turn and by additive scoring with total stability weights of 0.25, 0.50, and 0.75 (Supplementary Tables S1 and S2).

### 2.4. Hardened-State Characterization

Six hardened samples, X1–X3 and Y1–Y3, were characterized. X1/G-01 and Y3/S-03 were the material-scale candidates; X2/G-02 and Y2/S-02 were their respective references. X3/G-03 and Y1/S-01 provided additional within-system comparisons. Complete SEM and FTIR records were retained for all six samples, whereas XRD spectra and candidate–reference discussions focused on X1, X2, Y2, and Y3. Together, the techniques described morphology, the crystalline-phase framework, and bonding characteristics.

Morphology was examined with a ZEISS GeminiSEM 360 scanning electron microscope (Carl Zeiss Microscopy GmbH, Jena, Germany) at 5000× magnification. A 2 μm scale bar was retained in each image. Comparisons focused on the spatial relationships among hydration-product coverage, pores, particle connections, and needle-like features. Morphological interpretations of phases and bonding were considered alongside XRD and FTIR evidence [24].

Phase analysis was performed using a Rigaku Ultima IV X-ray diffractometer (Rigaku Corporation, Akishima, Japan) with Cu Kα radiation (λ=1.5406 Å), operated at 36 kV and 36 mA. Diffraction patterns were collected over a 2θ range of 5–80°, with a step size of 0.02° and a counting time of 1 s per step. The patterns were displayed on a common intensity scale with constant vertical offsets, without smoothing, peak fitting, or quantitative phase refinement. Assignment of low-angle AFt-related features followed published work on the ettringite crystal structure [25].

Functional groups were characterized using a Thermo Scientific Nicolet iS5 Fourier-transform infrared spectrometer (Thermo Electron Scientific Instruments LLC, Madison, WI, USA) in transmission mode. Spectra were acquired over approximately 400–4000 ${\mathrm{cm}}^{-1}$ at a spectral resolution of 4 ${\mathrm{cm}}^{-1}$, with 32 scans for both sample and background measurements. For comparison, the spectra were displayed over 500–4000 ${\mathrm{cm}}^{-1}$ with constant vertical offsets. O–H, bound-water, carbonate, and Si–O-related bands were identified using assignments from infrared studies of cement hydration [26].

### 2.5. Confined-Sand Validation

The sand was predominantly 0.25–2 mm in particle size. Specimens were prepared to a target relative density of approximately 63.15% and water content of 19.7%; the reported sand-source void ratio was approximately 0.53. Basic properties were determined according to GB/T 50123 [27]. The model box had internal dimensions of 0.5 m × 0.5 m × 0.5 m. The loading plate was 50 mm thick, and initial lateral confinement was 14.78 kPa. The injection pipe was embedded centrally, with its outlet 250 mm from each of the six boundary faces.

The confined-grouting apparatus and soil-pressure monitoring approach were previously used by our group to observe grouting-induced stresses under controlled confinement [28]. The present tests used this platform with different grout formulations, host-medium conditions, and injection parameters.

One confined-sand specimen was tested per condition. The model box was assembled and its bolts tightened before reconstituted sand at 19.7% water content was placed in 100 mm layers. Each layer was tamped uniformly, using the same filling and tamping procedure to target a relative density of approximately 63.15%. The pipe was embedded when the fill reached 250 mm, and filling continued to 500 mm using the same procedure. The outlet was thus located at the box center. After filling, three side panels were removed, and the specimen was transferred carefully to the loading system. Lateral confinement of 14.78 kPa was applied and allowed to stabilize before Stage I. After each condition, the specimen was unloaded and removed, and a new specimen was prepared for the next condition.

Grout was delivered by an XLKT-M25 self-priming grouting machine (Yongkang Carter Waterproof Plugging Equipment Factory, Yongkang, China) with a rated power of 3.6 kW, using a 5 m long, 10 mm diameter pipe. Input was controlled by the change in liquid level in a graduated supply bucket and elapsed time.

Each condition used 3.0 L of premixed grout, delivered in three 1.0 L stages. Each injection lasted 100 s, giving a manually controlled stage-averaged volumetric flow rate of

$$\bar{q}=\frac{\Delta V}{\Delta t}=\frac{1.0\;L}{100\;s}=0.010\;L\;s^{-1}=0.60\;L\;\min^{-1}.$$

This corresponds to 10 mL s^−1^ (600 mL $\min^{-1}$). Each injection was followed by a 300 s holding period before the next stage. After the third injection, the specimen was held until the soil-pressure response approached stability, with a nominal holding duration of up to 300 s before unloading. The cumulative injection time was 300 s, and the total volume was 3.0 L per condition. The nominal full protocol therefore lasted up to 1200 s from the first injection to the end of the final holding period. The value 0.60 L $\min^{-1}$ is the stage average calculated from volume and duration.

Monitoring used DMTY earth pressure cells (Nanjing Danmo Electronic Technology Co., Ltd., Nanjing, Jiangsu, China) measuring 20 mm × 5 mm with a 30 kPa range, connected to a DH3816N acquisition system (Donghua Testing Technology Co., Ltd., Jingjiang, Jiangsu, China) sampling at 2 Hz. Twenty cells were distributed across four faces, with five cells per face. Vertical lines 1#–5# and 11#–15# were on the front and rear faces, respectively; horizontal lines 6#–10# and 16#–20# were on the left and right faces. Sensor centers were 50, 150, 250, 350, and 450 mm from one boundary, at 100 mm intervals. Central, intermediate, and outer positions were approximately 250, 269, and 320 mm from the central outlet. Vertical cell 3# and horizontal cell 8# represented the centers of their respective monitoring faces for within-system comparisons among the three nominal W/B conditions.

For each system, the material candidate’s additive combination was retained to derive three injection conditions at nominal W/B=0.6, 0.7, and 0.8 (Table 3). Stages I and II corresponded to 100–500 s and 500–900 s, respectively; Stage III extended from 900 s to the end of the record. Nonoverlapping half-open intervals prevented duplicate assignment of observations at stage boundaries. The mean over the preceding 20 s defined the baseline; the mean over the final 20 s defined the stable value. The stage maximum defined the peak. For GFP–0.8, the third 1.0 L injection was completed before grout leakage invalidated the Stage III response. Its Stages I and II were retained, but Stage III was excluded from calculations. Stage volume and duration defined the controlled supply protocol.

### 2.6. Response Metrics and Criteria for the Preferred Condition

The peak stress increment and stable stress increment in stage *i* were defined by Equations (6) and (7):

(6) $$\Delta \sigma_{p,i}=\sigma_{p,i}-\sigma_{0,i},$$

(7) $$\Delta \sigma_{s,i}={\bar{\sigma }}_{s,i}-\sigma_{0,i}.$$

The signed stable-to-peak increment ratio was then defined by Equation (8):

(8) $$R_{i}=\frac{\Delta \sigma_{s,i}}{\Delta \sigma_{p,i}}\times 100\%.$$

Here, $\sigma_{0,i}$ is the pre-stage baseline, $\sigma_{p,i}$ is the stage peak, and ${\bar{\sigma }}_{s,i}$ is the final stable mean. When the peak and stable increments are both positive and have the same direction, $R_{i}$ is interpreted as the peak-to-stable retention ratio. A negative $R_{i}$ indicates a reversal of the final response relative to the stage peak or stress redistribution, rather than negative retention. The corresponding Stage I ratios were positive for all conditions and were used to compare nominal W/B values separately within GFP and SS-EA.

Stage I began at 100 s. Response preference was assessed from the joint magnitudes of the vertical and horizontal stable increments and retention ratios. Directional differences were described by $D_{\sigma }=\left|\Delta \sigma_{s,v}-\Delta \sigma_{s,h}\right|$ (kPa) and $D_{R}=\left|R_{v}-R_{h}\right|$ (percentage points, with *R* expressed in percent), interpreted alongside both directional amplitudes. Window sensitivity used lengths L=10, 20, and 40 s, with baseline [100−L,100) and final window [500−L,500) s; the Stage I peak interval remained [100,500) s. Each complete window contained 2L observations. Final morphology was described from visible segmentation, breakage, and local necking in the recovered ruler views after unloading at the end of the staged protocol. Continuity was qualitative. Stages II and III described cumulative responses within each test.

## 3. Results and Discussion

### 3.1. Effect of Nominal Water-to-Binder Ratio on Fresh-State Stability

**Figure 2:**
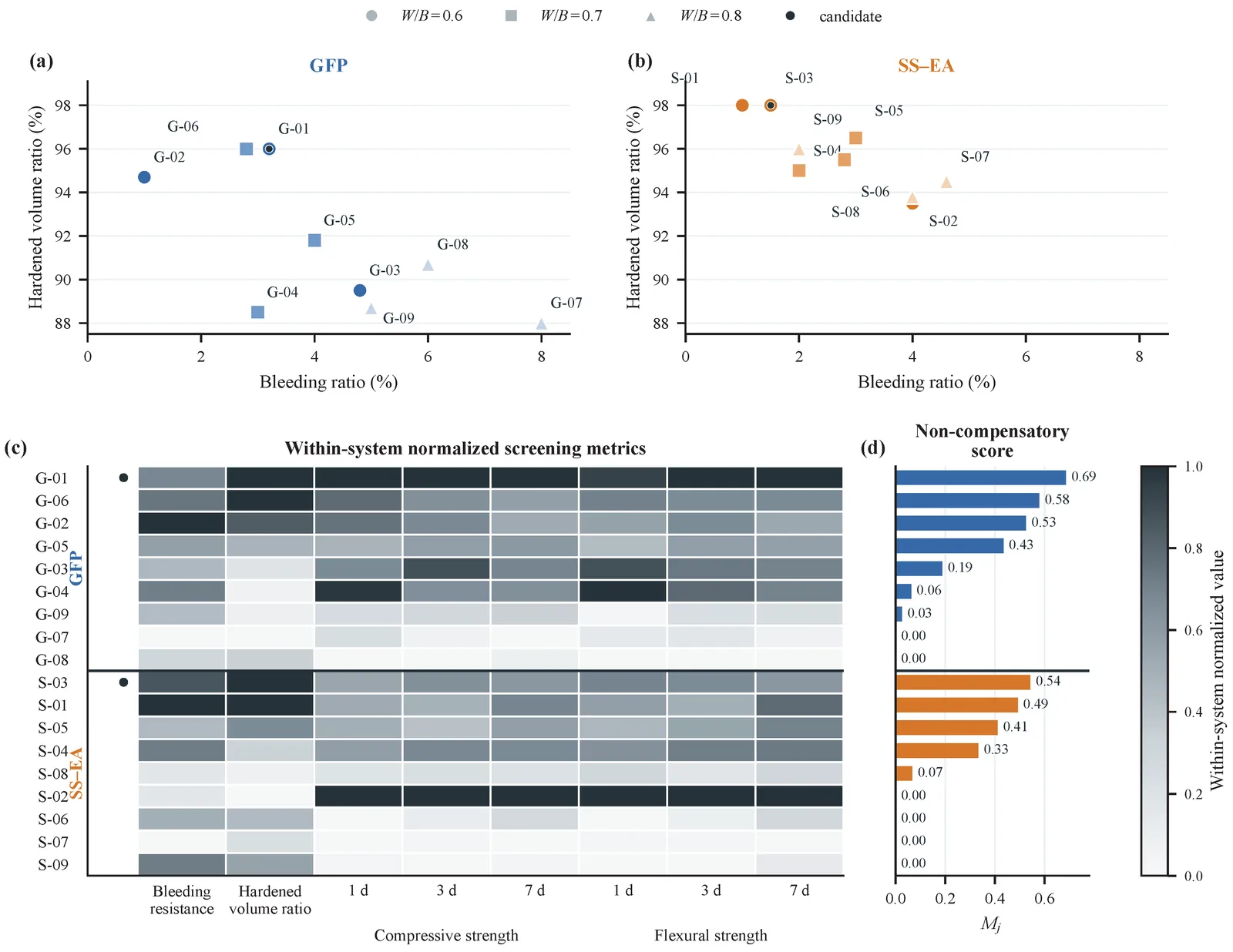
Material-scale candidate screening. Bleeding ratio versus hardened volume ratio for (**a**) GFP and (**b**) SS-EA. Both panels use the same axis limits. Marker shapes distinguish nominal W/B within each system, and small solid black dots identify the candidates. (**c**) Bleeding resistance, hardened volume ratio, and compressive and flexural strengths at 1, 3, and 7 d, normalized separately within GFP and SS-EA. The main-text heatmap uses color encoding without repeated cell values. (**d**) Noncompensatory scores $M_{j}$, aligned with the heatmap rows and labeled at the bar ends. Each system is ordered by $M_{j}$; small solid black dots identify G-01 and S-03. Colors indicate relative values within each system only. The fully annotated matrix is provided in Figure S4.

Under their respective original formulation conventions, both GFP and SS-EA showed declining fresh-state stability as nominal W/B increased (Figure 2). For GFP at nominal W/B=0.6, 0.7, and 0.8, mean bleeding ratios were 3.00%, 3.27%, and 6.33%, respectively, and mean hardened volume ratios were 93.40%, 92.10%, and 89.13%. The corresponding SS-EA bleeding ratios were 2.17%, 2.60%, and 3.53%, and hardened volume ratios were 96.50%, 95.67%, and 94.77%. Within each system, the group means at higher nominal W/B showed more bleeding and lower hardened volume retention. These groups also differ in additive composition, so the averages describe the tested design space rather than an isolated water-content effect. Supplementary cementitious materials and silicate components also influence rheology, bleeding, and hardening [2,10]. Within both design spaces, the nominal W/B=0.6 group had the lowest mean bleeding ratio and highest mean hardened volume ratio.

Fresh-state stability favored nominal W/B=0.6 within both design spaces. However, the final candidates required a joint assessment incorporating compressive and flexural strengths at 1, 3, and 7 d.

### 3.2. Strength Development and Balanced Material-Scale Selection

**Figure 3:**
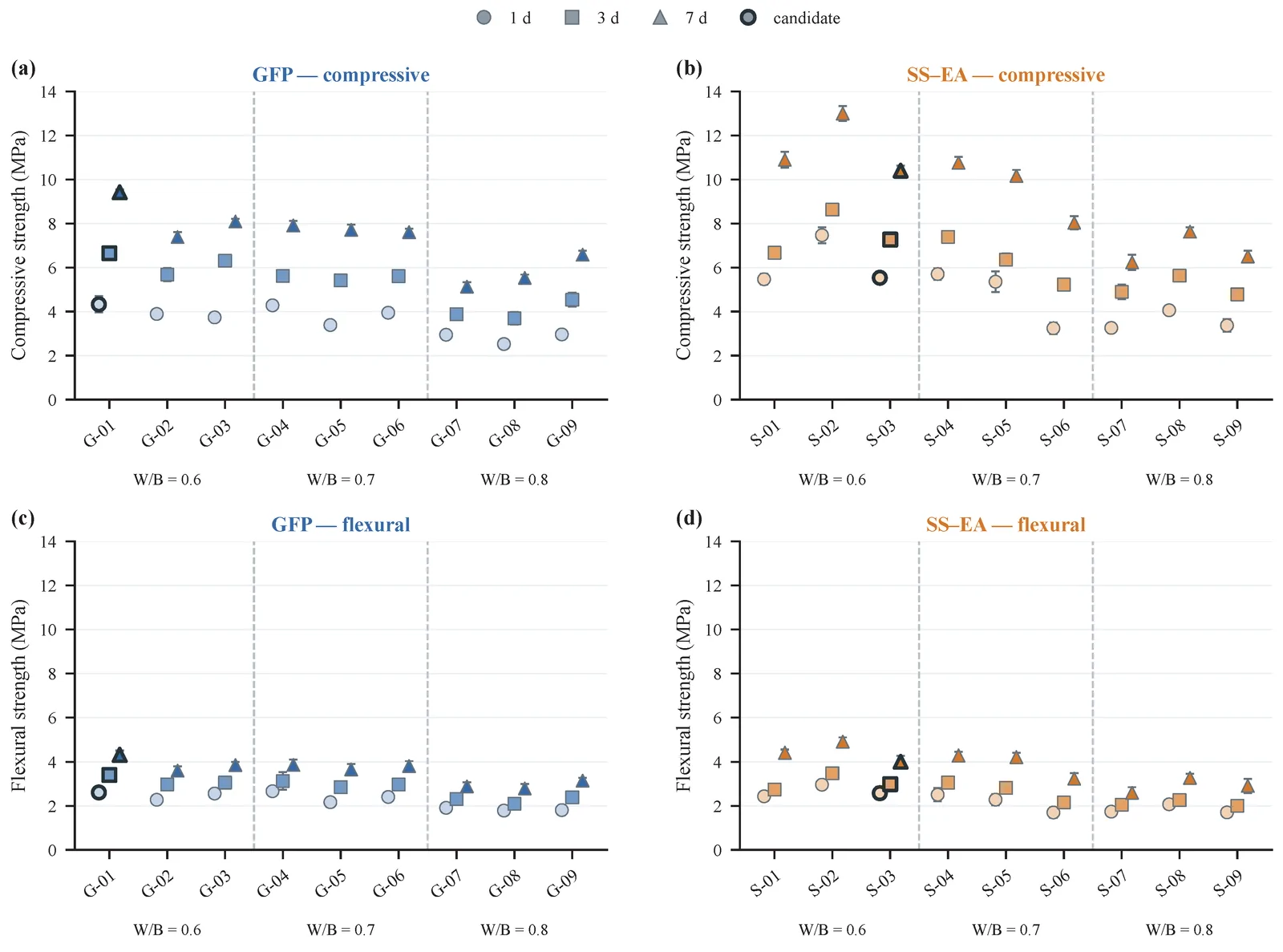
Mean compressive and flexural strengths of the 18 mixes. Compressive strengths of (**a**) GFP and (**b**) SS-EA, and flexural strengths of (**c**) GFP and (**d**) SS-EA. Circles, squares, and triangles denote 1, 3, and 7 d, respectively. Dashed lines separate nominal W/B=0.6, 0.7, and 0.8 within each system. All panels use a 0–14 MPa vertical scale. G-01 and S-03 are identified as candidates only by thicker, darker marker outlines. Points and error bars show the arithmetic mean ± sample SD of three specimens from the same preparation batch (n=3). Each compressive value represents one selected half-prism per parent prism.

Compressive and flexural strengths increased with age for both systems and generally decreased as their respective nominal W/B values increased (Figure 3). Increasing separately added mixing water may alter placement behavior but reduces the hardened load-bearing margin. Within each nominal W/B group, additive combinations also produced distinct mix rankings.

#### 3.2.1. Compressive Strength

For GFP, the mean 7 d compressive strengths at nominal W/B=0.6, 0.7, and 0.8 were 8.30, 7.75, and 5.75 MPa, respectively. The corresponding SS-EA values were 11.43, 9.65, and 6.79 MPa. G-01 had the highest GFP strength at all three ages, reaching 4.33, 6.65, and 9.42 MPa at 1, 3, and 7 d. S-02 defined the upper strength envelope for SS-EA, with a 7 d compressive strength of 13.00 MPa. S-03 increased continuously from 5.54 MPa at 1 d to 10.40 MPa at 7 d.

The decline in group-mean strength with increasing nominal W/B within each system is consistent with established effects on workability and hardened load-bearing capacity [7,8]. The performance of G-01 and nonmonotonic differences within GFP groups at the same nominal W/B indicate combined effects of GFP and PFA on strength development [19,29]. Differences between S-02 and S-03 are likewise consistent with the joint influence of SS and UEA on setting, volume retention, and hardened properties [10,20]. Nevertheless, maximum compressive strength represents only one load-bearing advantage and cannot replace joint constraints on bleeding, hardened volume ratio, and flexural strength.

#### 3.2.2. Flexural Strength and Balanced Candidate Screening

The mean 7 d flexural strengths for GFP at nominal W/B=0.6, 0.7, and 0.8 were 3.92, 3.78, and 2.94 MPa, respectively. The corresponding SS-EA values were 4.44, 3.91, and 2.93 MPa. G-01 increased from 2.61 MPa at 1 d to 4.31 MPa at 7 d. S-02 had the highest SS-EA flexural strength at all three ages, reaching 4.91 MPa at 7 d; S-03 increased from 2.58 to 4.00 MPa. The method of introducing sodium silicate can alter Portland cement setting and strength development [30]. Flexural capacity in SS-EA therefore depends on the component combination, rather than its nominal W/B alone.

At 7 d, S-02 reached 13.00 MPa in compression and 4.91 MPa in flexure, compared with 10.40 and 4.00 MPa for S-03. S-03 combined this lower strength with reduced bleeding (1.5% versus 4.0%) and greater hardened volume retention (98.0% versus 93.5%), illustrating the material-property trade-off [11]. The eight-indicator maximin rule selected G-01 and S-03 ($M_{j}=0.69$ and 0.54), both at nominal W/B=0.6. G-01 remained selected under all checked rules. For SS-EA, omitting hardened volume ratio selected S-04 (W/B=0.7), whereas additive weighting selected S-02 or S-01 (both W/B=0.6), showing that the candidate composition depends on the screening rule (Supplementary Tables S1 and S2).

### 3.3. Hardened-State Characterization of Selected and Reference Mixes

Samples X1–X3 and Y1–Y3 represented the final hardened states of the two systems. X1/G-01 and Y3/S-03 were the material-scale candidates, and X2/G-02 and Y2/S-02 were their respective references. X3/G-03 and Y1/S-01 provided additional within-system context. SEM, XRD, and FTIR assessed consistency between hardened structure and material-property rankings through morphology, crystalline phases, and bonding environments.

#### 3.3.1. SEM Morphology

**Figure 4:**
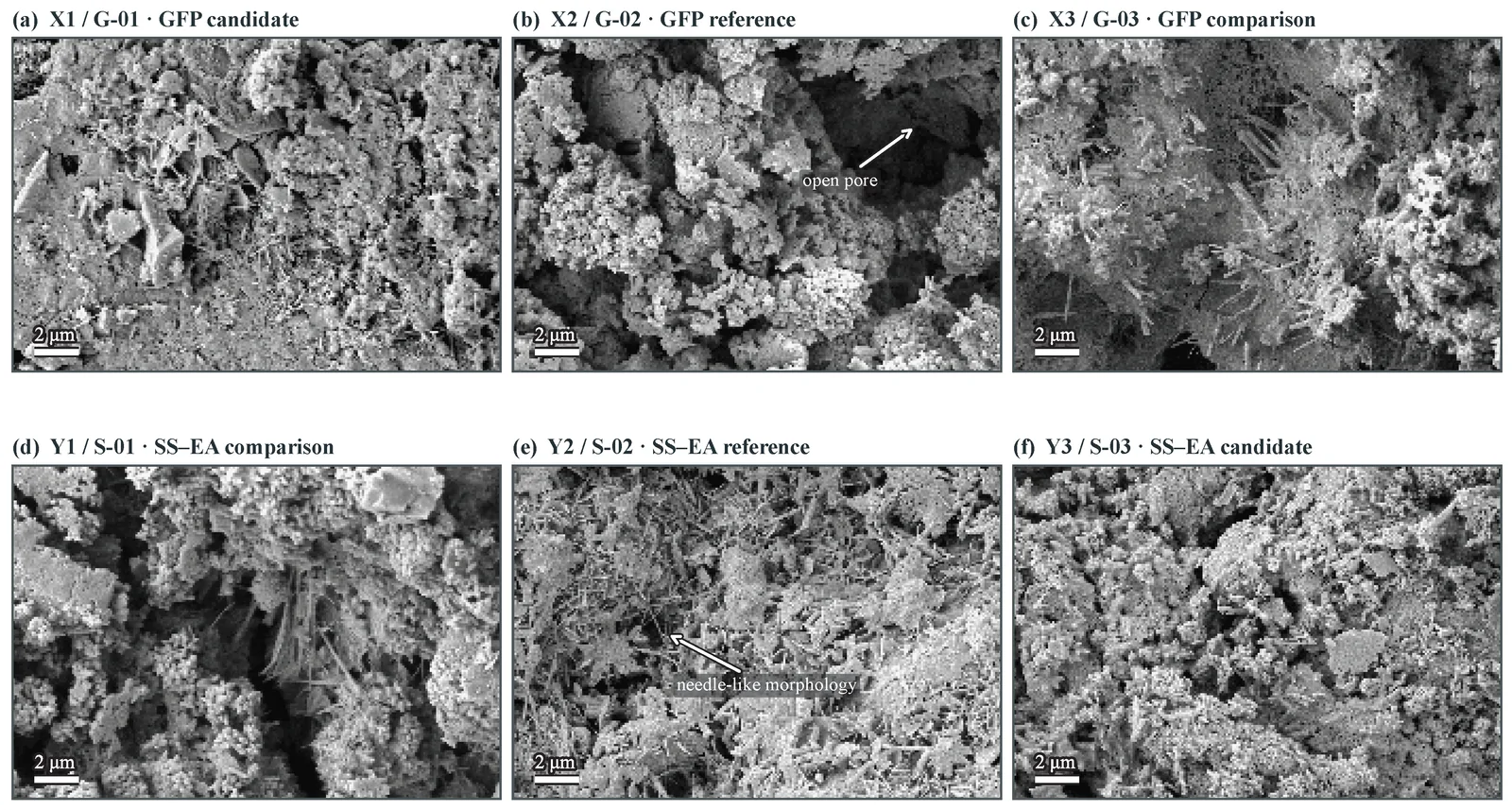
Final hardened-state SEM morphology of six mixes. Panels (**a**–**f**) show X1/G-01 (GFP candidate), X2/G-02 (GFP reference), X3/G-03 (additional GFP comparison), Y1/S-01 (additional SS-EA comparison), Y2/S-02 (SS-EA reference), and Y3/S-03 (SS-EA candidate), respectively. All panels show 5.00 K× SEM fields with 2 μm scale bars. Arrows identify open pores and needle-like features only in X2 and Y2.

For GFP (Figure 4), the X1 field contained layered, bundled needle-like, and flocculent features, with relatively continuous hydration-product coverage and connections between adjacent particles. X2 retained more angular particles with clearly defined outlines and more open pores. Cement hydration products commonly coexist at the micrometer scale [24], and previous GFP studies have shown compositional effects on hardened organization [19]. The more continuous coverage and connections in X1 were therefore consistent with its higher compressive and flexural strengths relative to X2.

For SS-EA, Y2 contained more interwoven needle bundles, with locally connected pores still visible. In Y3, needle- and rod-like features were more dispersed within a continuous matrix, with more complete coverage of particles and local pores. Previous SS-EA studies have linked compositional changes to setting and hardened organization [10,20]. The final morphology of Y3 was consistent with its low bleeding, high hardened volume ratio, and continued strength development. XRD features further constrained the assignment of the needle-like structures.

X3 and Y1 illustrated morphological variation within each system. SEM complemented the joint fresh-state and mechanical assessment through candidate–reference comparisons of hardened-state structure.

#### 3.3.2. XRD Phase Features

**Figure 5:**
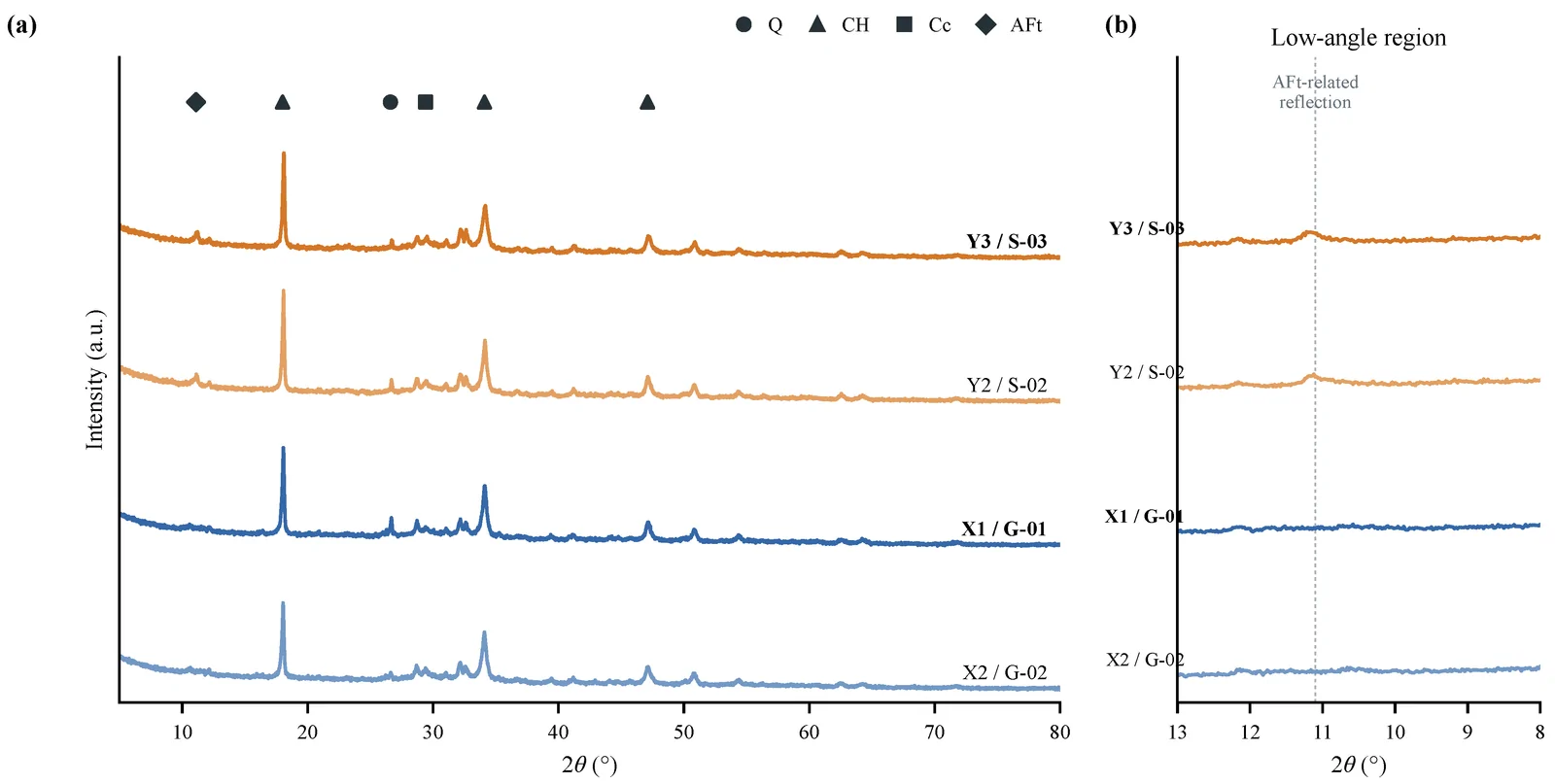
XRD patterns of the candidates and their corresponding references. (**a**) Full patterns from 5° to 80° for X1/G-01, X2/G-02, Y2/S-02, and Y3/S-03. (**b**) Enlarged AFt-related low-angle region from 8° to 13°. Q, CH, Cc, and AFt denote quartz, portlandite, calcite, and AFt-related phase markers. A thin gray dashed line marks the AFt-related reflection near 11.1°. The diffraction patterns are shown on a common intensity scale with constant vertical offsets. Phase markers indicate qualitative assignments; display processing is described in Section 2.4.

All four samples showed a common crystalline-phase framework (Figure 5) characteristic of hardened cementitious systems. Reflections near 18.0°, 34.1°, and 47.1° were associated with CH, and the strong peak near 26.6° arose mainly from crystalline SiO_2_. Carbonate-related responses occurred near 29.4° and in the adjacent region. A broad, poorly crystalline background over 20–35° reflected contributions from silicate hydration products and incompletely crystallized constituents. Y2 and Y3 showed AFt-related low-angle features near 11°. Together with the needle- and rod-like SEM features, these supported the presence of AFt-related structures [25].

Candidates and references shared the basic phase framework, with differences mainly in the relative prominence of characteristic reflections and the poorly crystalline background. These differences, together with SEM observations of coverage, pores, and particle connections, provided hardened-state support for the material-scale candidates.

#### 3.3.3. FTIR Bonding Features

**Figure 6:**
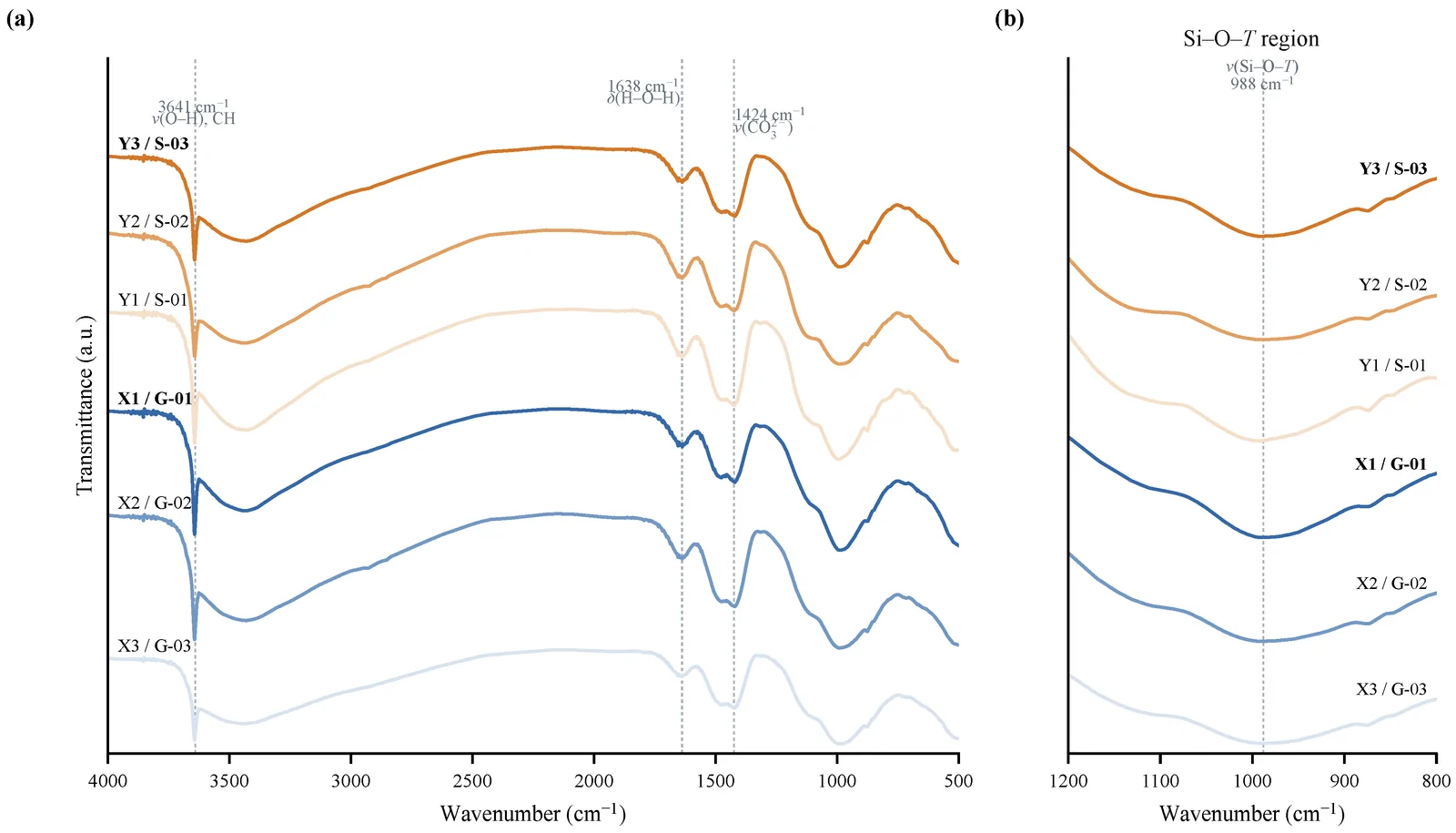
FTIR spectra of six mixes. (**a**) Full spectra of X1–X3 and Y1–Y3 over 4000–500 ${\mathrm{cm}}^{-1}$; thin gray dashed lines mark the principal bands at 3641, 1638, and 1424 ${\mathrm{cm}}^{-1}$. (**b**) Enlarged Si–O–*T* region over 1200–800 ${\mathrm{cm}}^{-1}$, with a thin gray dashed reference line near 988 ${\mathrm{cm}}^{-1}$; *T* denotes Si or Al. In both panels, the bottom-to-top order is X3/G-03, X2/G-02, X1/G-01, Y1/S-01, Y2/S-02, and Y3/S-03. Bold direct labels identify the candidates. Each transmittance spectrum was shifted by subtracting its full-spectrum minimum and applying constant 30 a.u. vertical spacing. Display processing is described in Section 2.4.

All samples showed principal absorption bands (Figure 6) near 3641, 1638, 1424, and 988 ${\mathrm{cm}}^{-1}$. These were assigned to CH-related O–H stretching, bound-water H–O–H bending, carbonate groups, and silicate-network Si–O–*T* vibrations, respectively, where *T* denotes Si or Al [26]. Similar overall band positions indicated a shared cementitious bonding framework among candidates and references. The main band within 1200–800 ${\mathrm{cm}}^{-1}$ lay near 988 ${\mathrm{cm}}^{-1}$ in all samples. Differences in shape and width were consistent with changes in local bonding environments associated with GFP, SS, and UEA.

Together, the SEM, XRD, and FTIR observations were consistent with the material-scale selection of G-01 and S-03. The central grouting question remained whether those rankings would persist after injection into confined granular media.

### 3.4. Responses and Ranking Changes in Confined-Sand Validation

All six direct-grouting conditions used the same three-stage volume–time-controlled injection protocol: 1.0 L in 100 s per stage, with a manually controlled stage-averaged volumetric flow rate of 0.60 L $\min^{-1}$ and 3.0 L per condition. Within-system rankings were based on Stage I, following the comparison boundaries in Section 2.1.

#### 3.4.1. Stage I Stress Development and Retention

**Figure 7:**
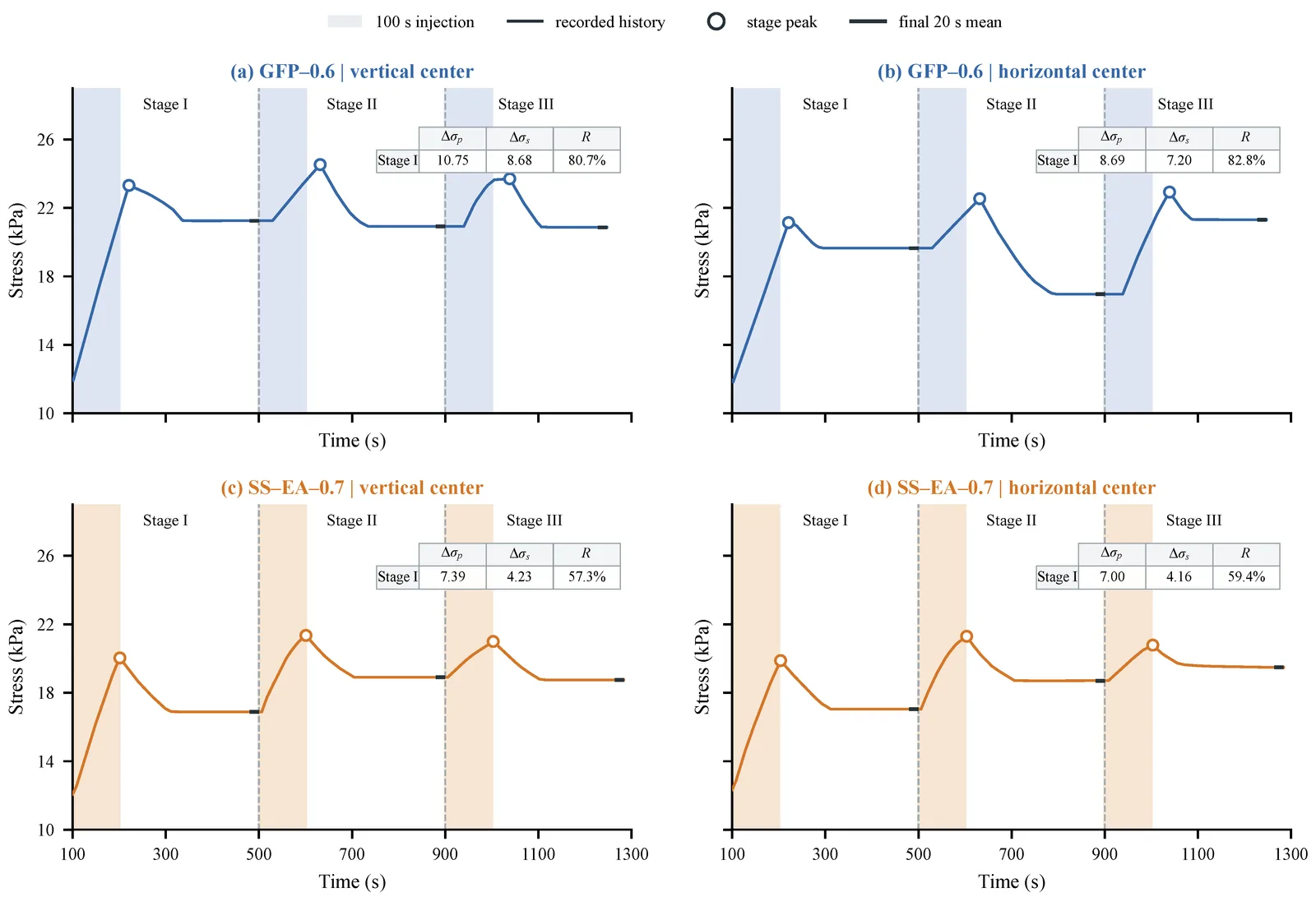
Recorded stress histories at the central cells on representative monitoring faces (vertical 3# and horizontal 8#) for the two preferred conditions. (**a**,**b**) Vertical and horizontal responses of GFP–0.6. (**c**,**d**) Vertical and horizontal responses of SS-EA–0.7. Shaded regions denote the 1.0 L/100 s injection periods. Open circles mark stage peaks; short horizontal lines mark stable means over the final 20 s. Insets list Stage I peak increments $\Delta \sigma_{p}$, stable increments $\Delta \sigma_{s}$ (kPa), and retention ratios *R*. All panels share horizontal and vertical ranges of 100–1300 s and 10–29 kPa. Lines connect the unsmoothed 2 Hz observations.

The central stress histories in Figure 7 showed a rapid rise during injection, a peak, post-peak relaxation, and stabilization during holding. Stage I provided the within-system comparison, while Stages II and III described cumulative responses of the grout–sand structure.

**Figure 8:**
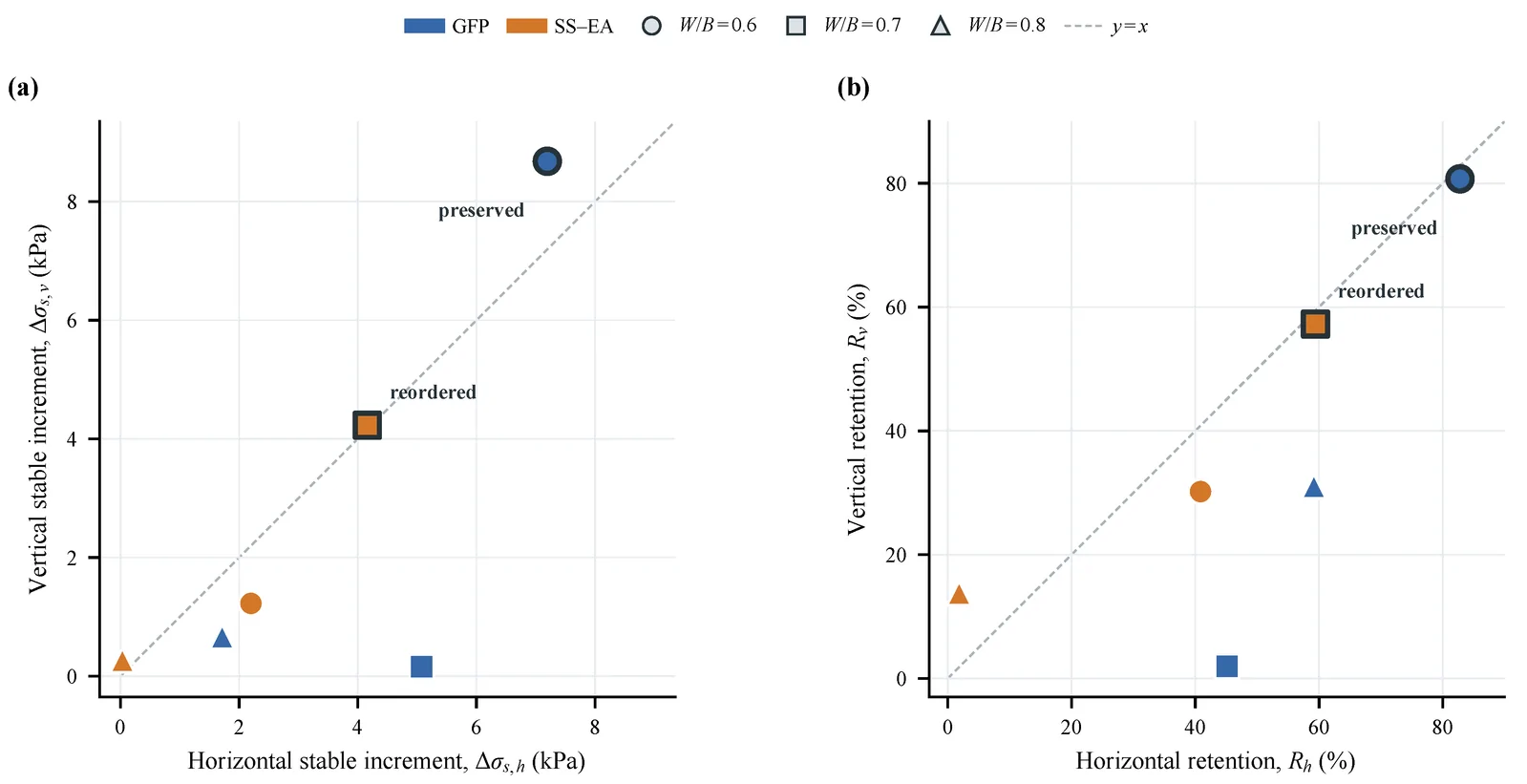
Magnitude and directional balance of Stage I central responses for the six conditions (vertical 3# and horizontal 8#). (**a**) Horizontal stable stress increment $\Delta \sigma_{s,h}$ versus vertical stable stress increment $\Delta \sigma_{s,v}$. (**b**) Horizontal retention ratio $R_{h}$ versus vertical retention ratio $R_{v}$. Each point represents one condition. Blue and orange denote GFP and SS-EA; circles, squares, and triangles indicate nominal W/B=0.6, 0.7, and 0.8 within each system. The thin gray dashed line y=x indicates equal directional values. Upper-right positions indicate larger responses in both directions; deviation from the line indicates directional differences. GFP–0.6 and SS-EA–0.7 have slightly thicker black outlines and are labeled “preserved” and “reordered,” respectively. All points represent Stage I; grout leakage in GFP–0.8 occurred during Stage III.

For GFP–0.6, the Stage I horizontal–vertical stable-response coordinates were (7.20, 8.68) kPa, and the retention coordinates were (82.8%, 80.7%). For SS-EA–0.7, the corresponding coordinates were (4.16, 4.23) kPa and (59.4%, 57.3%). Figure 8 places horizontal and vertical responses on the horizontal and vertical axes, respectively, to evaluate magnitude and directional balance together. Movement toward the upper right indicates increases in both directions; proximity to y=x indicates a smaller vertical–horizontal difference. Complete Stage I central histories are shown in Figure S2; Stage II–III stable increments and signed ratios are provided in Figure S1.

Among the three GFP conditions, GFP–0.6 occupied the upper-right region in both the stable-increment and retention planes. Horizontal and vertical stable increments differed by 1.48 kPa, while $R_{h}$ and $R_{v}$ differed by only 2.1 percentage points. Relatively high response magnitude therefore coincided with directional balance. GFP–0.7 had a horizontal stable increment of 5.08 kPa but a vertical increment of only 0.16 kPa, placing it well away from the diagonal. GFP–0.8 occupied the low-magnitude region. These results indicate ranking preservation for the GFP material candidate at W/B=0.6.

Among the SS-EA conditions, SS-EA–0.7 lay closest to the upper-right region in the stable-increment plane. Its horizontal and vertical stable increments differed by only 0.07 kPa, and its retention ratios differed by 2.2 percentage points. SS-EA–0.6 and SS-EA–0.8 had lower magnitudes in both directions or larger directional differences in retention. Proximity to y=x alone was insufficient for selection; response magnitude also had to be considered. The preferred SS-EA condition therefore underwent ranking reordering from W/B=0.6 at the material scale to W/B=0.7 in confined sand. Table 4 summarizes the central responses and recovered grout body evidence.

The 10, 20, and 40 s windows retained GFP–0.6 and SS-EA–0.7 as the highest recorded conditions for all four directional stress metrics (Supplementary Table S4). At 40 s, the vertical increment of GFP–0.7 and both increments of SS-EA–0.8 became negative relative to their baselines. The signs of these small responses therefore depend on the baseline window, even though the preferred conditions remain unchanged.

#### 3.4.2. Recovered Grout Body Morphology and Spatial Continuity

**Figure 9:**
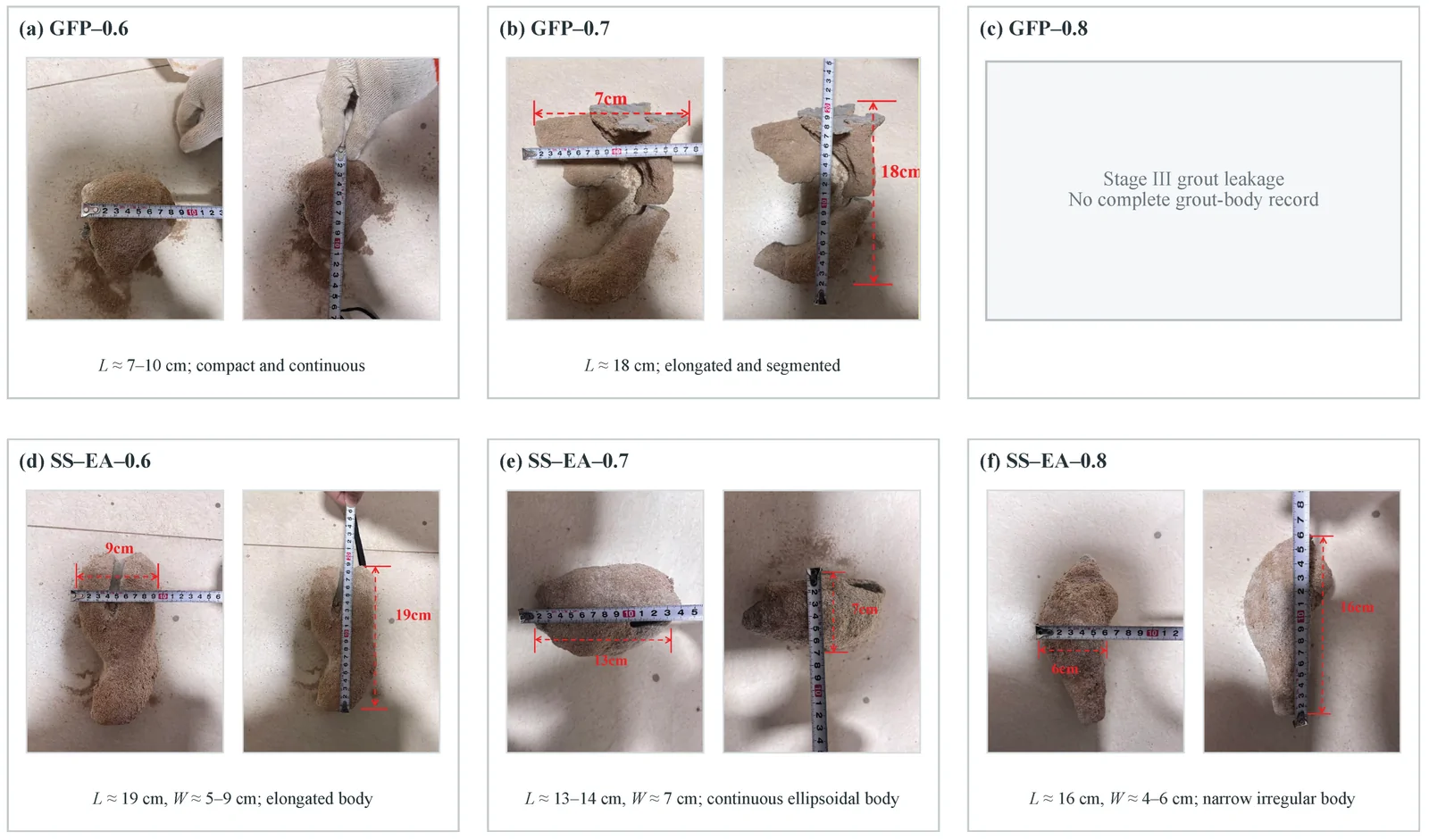
Representative recovered grout bodies for (**a**) GFP–0.6, (**b**) GFP–0.7, (**c**) GFP–0.8, (**d**) SS-EA–0.6, (**e**) SS-EA–0.7, and (**f**) SS-EA–0.8. Two ruler-measurement views document final morphology after the staged protocol and unloading for each of the five conditions with complete recovered bodies. Principal dimensions and morphological features are given below the panels. GFP–0.8 experienced grout leakage during Stage III, and no complete body was recovered. Measurement records from all viewing directions are provided in Figure S3.

GFP–0.6 formed a concentrated, continuous body approximately 7–10 cm in size (Figure 9). At nominal W/B=0.7, the body extended approximately 18 cm along a preferential direction, with local narrowing and an elongated, segmented form. GFP–0.8 leaked during Stage III and did not yield a completely recoverable body.

SS-EA–0.6 formed an elongated body approximately 19 cm along its long axis and 5–9 cm wide. SS-EA–0.7 formed a continuous ellipsoidal body approximately 13–14 cm × 7 cm, with greater lateral spread. SS-EA–0.8 formed an irregular slender body approximately 16 cm × 4–6 cm. These observations complemented the Stage I stress responses through evidence of spatial spread and continuity.

### 3.5. Cementitious Material–Medium Performance Transferability

#### 3.5.1. From Material Properties to Performance in a Host Medium

Material-scale and medium-specific assessments describe different parts of cementitious material performance. Here, performance transferability refers to the preservation of within-system mix preference across these two assessments. The former identifies candidates without pronounced weaknesses in fresh-state stability, hardened strength, and final structure. The latter compares stress development and retention during the recorded holding period, with final grout body morphology providing spatial context under the specified sand, confinement, and supply conditions. Grout particle–pore throat compatibility, filtration, and medium gradation govern groutability and penetration [15,16,17,18]. Setting progression and local grout–particle interactions further influence spreading morphology and stress propagation [31,32]. Thus, fresh-state stability and hardened load-bearing properties must be considered alongside the material’s spatial continuity and mechanical response in its host medium.

**Figure 10:**
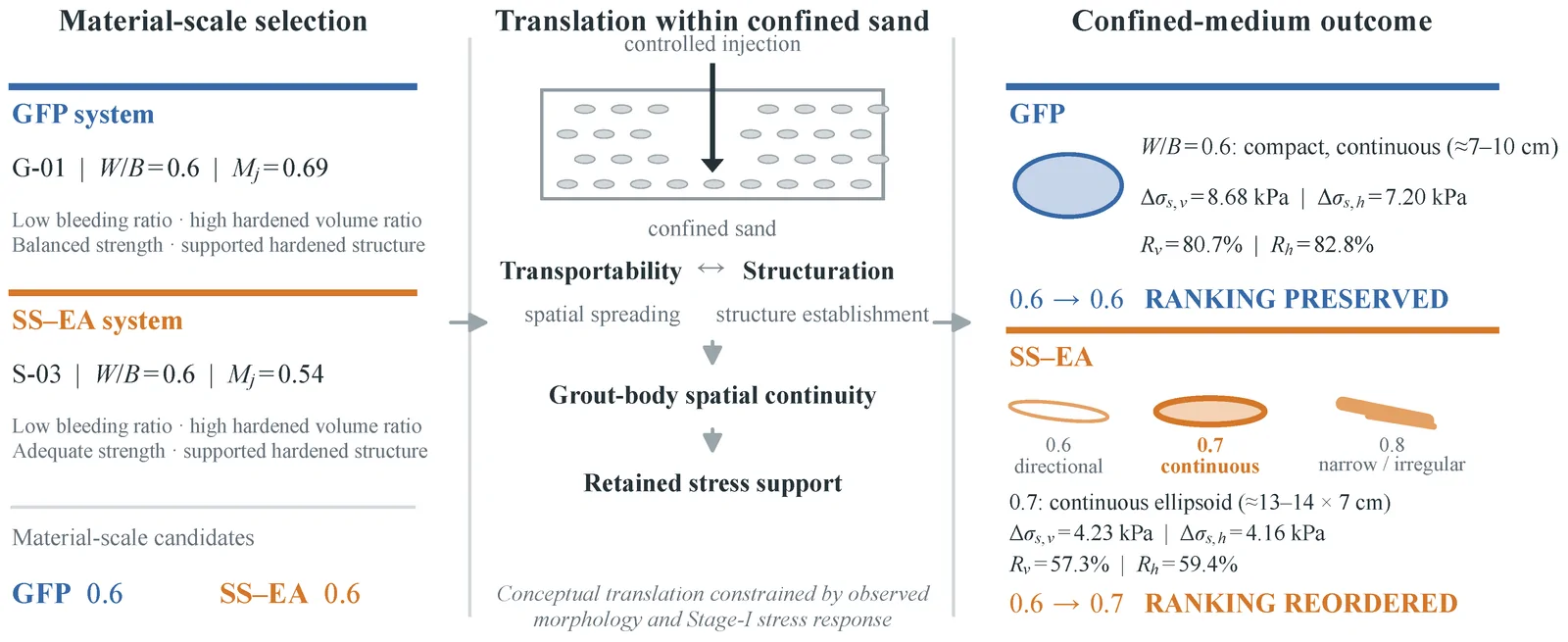
Interpretive framework linking material-scale screening to responses in confined sand. The left column represents material-scale screening. The middle column organizes the conceptual transition through transportability, structuration, grout body spatial continuity, and stress support. The right column summarizes observed evidence, including recovered grout bodies, Stage I stable stress increments, and retention ratios. Transportability and structuration are proposed interpretations of the stress and final-morphology observations.

Here, “transportability” describes the relative tendency for spatial spread under the current supply protocol. “Structuration” describes the relative tendency to form a continuous body and maintain stress after spreading. Their conceptual balance connects to the observations through spatial spread, grout body continuity, and stress support. Figure 10 summarizes this interpretation while distinguishing conceptual processes from directly observed evidence.

#### 3.5.2. Relative Balance Between Transportability and Structuration

The following interpretation concerns the fixed-additive sequences within each system. Confinement can alter grout body morphology and soil response [33]. The effect of sodium silicate on cement setting depends on composition and its introduction method [30,34]; in this study, SS was introduced with the mixing water. Rheological recovery and strength development can also be coupled [35,36]. Together, the observed morphology and stress patterns are consistent with a transportability–structuration balance.

At the material scale, G-01 combined low bleeding, a high hardened volume ratio, balanced strengths, and consistent hardened-state structure. In sand, its concentrated, continuous body coincided with a relatively balanced stress response in both directions. These observations are consistent with preservation of its preferred nominal W/B for stress retention under the tested supply and confinement conditions.

For SS-EA, S-03 provided the best noncompensatory balance among bleeding, hardened volume ratio, and strength at the material scale. In confined sand, however, formulation differences were first expressed through different patterns of spatial spread. Compared with the elongated form at nominal W/B=0.6 and the irregular slender form at 0.8, the body at 0.7 was more continuous and spread more fully laterally. It also accompanied a more balanced stress response in both directions. This combination is consistent with a more favorable transportability–structuration balance at nominal W/B=0.7, despite material-scale selection at 0.6.

#### 3.5.3. Implications for Cementitious Material Selection and Limits of Applicability

The results support a two-stage approach to cementitious grout selection based on material properties and their expression in a host medium. Material-scale candidates are first identified by noncompensatory assessment of bleeding ratio, hardened volume ratio, and age-dependent strengths. Rankings are then reassessed in representative host media under defined placement and confinement conditions. Stable stress increments, retention ratios, and recovered grout body continuity provide complementary evidence for this validation. Application-specific selection still requires recalibration for scale, equipment capacity, and staged-injection objectives. For compensation or corrective grouting, these considerations link material selection with the spatial spread and controlled stress development required by the application [5,6].

The confined-sand comparison comprised one independent test per condition, so between-specimen variability and statistical significance of the observed preferences were not determined. The platform has a published application [28], which provides methodological precedent rather than replication of the present conditions. Specimen-preparation and injection-history variability was not independently quantified. The proposed transportability–structuration balance offers an interpretation of the stress and final-morphology observations. Concurrent pump-pressure, rheological, filtration, setting-rate, and restrained-expansion measurements were not available. The pressure-cell records do not separate pore-pressure and solid-skeleton contributions. The reported retention covers post-injection holding periods of approximately 300 s, while material strengths cover 1–7 d. The observed preferences apply to the tested formulations, medium-dense sand, model scale, confinement, and staged supply. Longer-term reaction, volume change, groundwater exposure, and changing confinement require assessment over the intended service period.

## 4. Conclusions

1. This study connected material-scale screening by bleeding ratio, hardened volume ratio, and strength with confined-sand assessment of stress development, stress retention, and grout body continuity. The resulting two-stage evidence chain directly tested the transferability of material-scale selection.
2. Under the stated maximin rule, both systems selected nominal W/B=0.6 within their material design spaces. In the recorded confined-sand tests, GFP retained preference at nominal W/B=0.6, whereas SS-EA shifted to nominal W/B=0.7. Stage I stress responses in both directions, supported by final recovered morphology, provided mechanical and spatial evidence for these outcomes.
3. Under the tested conditions, material-scale properties alone did not uniquely predict the preferred confined-sand condition. Incorporating medium-dependent responses provides additional evidence for final mix selection after material-scale screening.

## Figures and Tables

**Table 1:** Principal chemical compositions and related chemical indices of the raw materials (mass fraction, %).

Chemical Index	OPC	PFA	GFP	UEA	SS
SiO_2_	20.98	–	77.14	–	26.98
Al_2_O_3_	4.72	–	1.85	–	–
Fe_2_O_3_	3.51	–	0.14	–	–
CaO	63.09	6.70	1.06	–	–
MgO	1.72	–	0.14	29.00	–
SO_3_	2.58	2.00	–	–	–
Na_2_O	–	–	18.34	–	8.53
Na_2_O_*eq*_	0.55	–	–	–	–
f-CaO	0.62	0.65	–	–	–
Cl^−^	0.015	0.01	–	–	–
TiO_2_	–	–	0.022	–	–
B_2_O_3_	–	–	0.62	–	–
LOI	2.15	4.13	0.59	–	–
Total alkalis	–	–	–	4.00	–

LOI: loss on ignition. A dash denotes an unavailable value; chemical indices are partial for PFA and UEA.

**Table 2:** The 18 material-scale mixes and corresponding microstructural samples.

System	Mix ID	W/B	$w/s_{\mathrm{calc}}$	PFA/%	GFP/%	UEA/%	SS/%	Micro. Sample
GFP	G-01	0.6	0.5970	10.0	0.5	–	–	X1
GFP	G-02	0.6	0.5911	15.0	1.5	–	–	X2
GFP	G-03	0.6	0.5941	20.0	1.0	–	–	X3
GFP	G-04	0.7	0.6897	10.0	1.5	–	–	–
GFP	G-05	0.7	0.6965	15.0	0.5	–	–	–
GFP	G-06	0.7	0.6931	20.0	1.0	–	–	–
GFP	G-07	0.8	0.7921	10.0	1.0	–	–	–
GFP	G-08	0.8	0.7882	15.0	1.5	–	–	–
GFP	G-09	0.8	0.7960	20.0	0.5	–	–	–
SS-EA	S-01	0.6	0.5587	5.0	–	4.0	2.0	Y1
SS-EA	S-02	0.6	0.5791	2.5	–	3.0	2.5	Y2
SS-EA	S-03	0.6	0.5394	7.5	–	5.0	1.5	Y3
SS-EA	S-04	0.7	0.6498	5.0	–	4.0	2.0	–
SS-EA	S-05	0.7	0.6279	7.5	–	5.0	1.5	–
SS-EA	S-06	0.7	0.6731	2.5	–	3.0	2.5	–
SS-EA	S-07	0.8	0.7410	5.0	–	4.0	2.0	–
SS-EA	S-08	0.8	0.7495	2.5	–	5.0	1.5	–
SS-EA	S-09	0.8	0.7327	7.5	–	3.0	2.5	–

Additive contents use OPC+PFA mass for GFP and OPC mass for SS-EA. Nominal W/B and calculated $w/s_{\mathrm{calc}}$ follow Section 2.2.2. The calculation assumes dry powders and 35.5% SS solution solids; full component accounting is in Supplementary Table S3. A dash denotes an inapplicable component or a mix not characterized microscopically.

**Table 3:** Test conditions for confined direct grouting.

Condition	W/B	$w/s_{\mathrm{calc}}$	PFA/%	GFP/%	UEA/%	SS/%	Lateral Pressure (kPa)	Record End (s)	Valid Stages
GFP–0.6	0.6	0.5970	10.0	0.5	–	–	14.78	1247.0	I–III
GFP–0.7	0.7	0.6965	10.0	0.5	–	–	14.78	1300.0	I–III
GFP–0.8	0.8	0.7960	10.0	0.5	–	–	14.78	1124.5	I–II; leakage in III
SS-EA–0.6	0.6	0.5394	7.5	–	5.0	1.5	14.78	1180.5	I–III
SS-EA–0.7	0.7	0.6279	7.5	–	5.0	1.5	14.78	1283.5	I–III
SS-EA–0.8	0.8	0.7163	7.5	–	5.0	1.5	14.78	1247.0	I–III

Record end denotes the endpoint on the acquisition time axis; Stage I began at 100 s. The final holding period ended when the soil-pressure response approached stability, with a nominal maximum of 300 s. W/B follows the system-specific nominal definitions in Section 2.2.2. A dash denotes an inapplicable component. For GFP–0.8, the third 1.0 L injection was completed before grout leakage invalidated the Stage III data, which were excluded from valid-stage analyses. The calculated $w/s_{\mathrm{calc}}$ assumes dry powders and accounts for water and solids in the SS solution.

**Table 4:** Stage I central responses and recovered grout body evidence for the six conditions.

Condition	Vertical $\Delta \sigma_{s}$ (kPa)	Vertical *R* (%)	Horiz. $\Delta \sigma_{s}$ (kPa)	Horiz. *R* (%)	Recovered Grout Body Evidence
GFP–0.6	8.68	80.7	7.20	82.8	Concentrated, continuous body, approximately 7–10 cm
GFP–0.7	0.16	1.9	5.08	45.1	Elongated, segmented body with a long axis of approximately 18 cm
GFP–0.8	0.67	31.1	1.71	59.2	Stage III grout leakage; no complete body recovered
SS-EA–0.6	1.23	30.2	2.20	40.9	Elongated body, approximately 19 cm long and 5–9 cm wide
SS-EA–0.7	4.23	57.3	4.16	59.4	Continuous ellipsoidal body, approximately 13–14×7 cm
SS-EA–0.8	0.27	13.8	0.03	1.8	Irregular slender body, approximately 16×4–6 cm

The vertical and horizontal central responses were measured by pressure cells 3# and 8#, respectively, each at the center of a five-cell line on its monitoring face. Retention ratios were calculated from unrounded increments. For GFP–0.8, the third 1.0 L injection was completed before grout leakage invalidated the Stage III data. Recovered-body descriptions refer to final post-test morphology after the staged protocol and unloading; continuity was assessed qualitatively from visible integrity, segmentation, and necking.

## Data Availability

The Supplementary Materials provide supporting formulation calculations, screening analyses, and processed stress comparisons. Additional data supporting the findings of this study are available from the corresponding author upon reasonable request.

## Supplementary Materials

The following supporting information can be downloaded at https://www.mdpi.com/article/10.3390/ma19214545/s1. Figure S1: Stage II–III central responses for the six conditions. (a,b) Stable stress increments at vertical cell 3# and horizontal cell 8#. (c,d) Corresponding signed stable-to-peak ratios. Blue and orange denote GFP and SS-EA; solid circles and open diamonds denote Stages II and III. Gray horizontal lines indicate y=0. Negative values indicate response reversal relative to the stage baseline or stress redistribution. GFP–0.8 has no valid Stage III result because of grout leakage. Dark gray crosses denote missing results only; their vertical positions do not encode a numerical value, particularly zero. Stage I selection metrics are shown in Figure 8 of the main text. Figure S2: Stage I central stress-increment histories for the six conditions. Panels (a–f) show GFP–0.6, GFP–0.7, GFP–0.8, SS-EA–0.6, SS-EA–0.7, and SS-EA–0.8, respectively. Solid and dashed lines denote vertical cell 3# and horizontal cell 8#. Neutral gray shading marks the 1.0 L/100 s injection period. Curves show increments in the 2 Hz records relative to the mean baseline over the preceding 20 s, without smoothing or interpolation. All panels use horizontal and vertical ranges of 100–500 s and −3.2–12.0 kPa. Gray horizontal lines indicate zero increment. Figure S3: Post-test ruler-measurement records after the staged protocol and unloading for (a) GFP–0.6, (b) GFP–0.7, (c) GFP–0.8, (d) SS-EA–0.6, (e) SS-EA–0.7, and (f) SS-EA–0.8. For the five recovered bodies, the available viewing directions, physical rulers, and dimension annotations are shown. GFP–0.8 has no complete grout body record because of Stage III grout leakage. Representative views from these records are shown in Figure 9 of the main text. Figure S4: Complete matrix of normalized material-screening indicators and noncompensatory scores. Values were normalized to 0–1 separately within GFP and SS-EA. The final column, $M_{j}$, is the minimum of the eight normalized indicators for each mix and is separated from them by a vertical line. The grayscale range is 0–1, and the row order matches Figure 2 of the main text. Cell values are shown to two decimal places. Small solid black dots identify the two candidates, G-01 and S-03. Table S1: Candidates under the primary and leave-one-indicator-out maximin rules. Table S2: Additive screening scores for the three prespecified stability weights. Bold values identify the highest score within each system and weighting rule. Table S3: Normalized component masses (g) and calculated water-to-solid ratios for 18 material mixes and six injection conditions. Table S4: Stage I window sensitivity of central stress increments, retention ratios, and directional differences. The 20 s rows are the primary analysis.

## References

[1] da Rocha Gomes S., Ferrara L., Sánchez L., Moreno M.S. (2023). A comprehensive review of cementitious grouts: Composition, properties, requirements and advanced performance. Constr. Build. Mater..

[2] Khayat K.H., Yahia A., Sayed M. (2008). Effect of supplementary cementitious materials on rheological properties, bleeding, and strength of structural grout. ACI Mater. J..

[3] Hu H., Lu Y., Deng C., Gan B., Xie Z., Cai Y., Chu A. (2024). Experimental study on grout-soil interaction effects in sandy soil under different water-to-cement ratios. Buildings.

[4] Han C., Wei J., Zhang W., Yang F., Yin H., Xie D., Xie C. (2022). Quantitative permeation grouting in sand layer with consideration of grout properties and medium characteristics. Constr. Build. Mater..

[5] Zhang D.M., Huang Z.K., Wang R.L., Yan J.Y., Zhang J. (2018). Grouting-based treatment of tunnel settlement: Practice in Shanghai. Tunn. Undergr. Space Technol..

[6] Zhou S., Xiao J., Di H., Zhu Y. (2018). Differential settlement remediation for new shield metro tunnel in soft soils using corrective grouting method: Case study. Can. Geotech. J..

[7] Chen X., Bi R., Zhang L. (2019). Effect of water-to-cement ratio on sulfo-aluminate type cementitious grouting materials. Mag. Concr. Res..

[8] Li H., Yang K., Guan X. (2020). Effect of water-to-binder ratio on the properties of CSA cement-based grouting materials with LiAl-LDH. Adv. Compos. Lett..

[9] Li S., Sha F., Liu R., Li Z., Zhang Q. (2017). Investigation of viscous behaviour and strength of microfine-cement-based grout mixed with microfine fly ash and superplasticiser. Adv. Cem. Res..

[10] Cavusoglu I., Yilmaz E., Yilmaz A.O. (2021). Sodium silicate effect on setting properties, strength behavior and microstructure of cemented coal fly ash backfill. Powder Technol..

[11] Li S., Zhang J., Li Z., Gao Y., Qi Y., Li H., Zhang Q. (2019). Investigation and practical application of a new cementitious anti-washout grouting material. Constr. Build. Mater..

[12] Mollamahmutoglu M., Avci E. (2015). Effectiveness of microfine Portland cement grouting on the strength and permeability of medium to fine sands. Period. Polytech. Civ. Eng..

[13] Li Z., Zhang L., Chu Y., Zhang Q. (2020). Research on influence of water-cement ratio on reinforcement effect for permeation grouting in sand layer. Adv. Mater. Sci. Eng..

[14] Cheng W.C., Ni J.C., Shen J.S., Wang Z.F. (2018). Modeling of permeation and fracturing grouting in sand: Laboratory investigations. J. Test. Eval..

[15] Eklund D., Stille H. (2008). Penetrability due to filtration tendency of cement-based grouts. Tunn. Undergr. Space Technol..

[16] Fraccica A., Spagnoli G., Romero E., Arroyo M., Gómez R. (2022). Permeation grouting of silt-sand mixtures. Transp. Geotech..

[17] Farhadian H., Maleki Z. (2023). Groutability classification of granular soils with cement grouts. J. Rock Mech. Geotech. Eng..

[18] Zhang H., Guo Y., Sun T., Liu T., Jiao Y., Li X. (2024). Investigation on the penetrability of cement slurries with three solid-phase particle sizes in coral sands with different gradations. Constr. Build. Mater..

[19] Li Q., Zhao G., Wang Y., Cheng X., Huang S., Li Y., Meng X. (2025). Experimental study on hydration products and engineering properties of glass fiber powder modified fly ash-cement composite grouting material. J. China Coal Soc..

[20] Cui H., Meng X., Cheng X., Zhao G., Gu Q., Wang Y., Zhu S. (2025). Research and test of sodium silicate-expansion agent-fly ash cement-based composite grouting material. China Min. Mag..

[21] (2023). Common Portland Cement.

[22] (2017). Fly Ash Used for Cement and Concrete.

[23] (2021). Test Method of Cement Mortar Strength (ISO Method).

[24] Famy C., Brough A.R., Taylor H.F.W. (2003). The C-S-H gel of Portland cement mortars: Part I. The interpretation of energy-dispersive X-ray microanalyses from scanning electron microscopy, with some observations on C-S-H, AFm and AFt phase compositions. Cem. Concr. Res..

[25] Colmenero F., Fernández A.M., Almendros-Ginestà O., Missana T. (2024). Density functional theory study of the crystal structure and infrared spectrum of a synthetized ettringite mineral. Minerals.

[26] Ylmén R., Wadsö L., Panas I. (2010). Insights into early hydration of Portland limestone cement from infrared spectroscopy and isothermal calorimetry. Cem. Concr. Res..

[27] (2019). Standard for Geotechnical Testing Method.

[28] Chen Q., Cheng W., Zou B., Kong B., Deng Y., Long X. (2025). Experimental study on additional stress induced by grouting with polyurethane-modified cementitious materials under confined conditions. J. Polym. Mater..

[29] Zhang C., Fu J., Song W. (2023). Mechanical model and strength development evolution of high content fly ash–cement grouting material. Constr. Build. Mater..

[30] Szewczenko W., Kotsay G. (2021). Influence of water glass introduction methods on selected properties of Portland cement. Materials.

[31] Zhang L., Yu R., Zhang Q., Liu R., Feng H., Chu Y. (2022). Permeation grouting diffusion mechanism of quick setting grout. Tunn. Undergr. Space Technol..

[32] Boschi K., di Prisco C.G., Ciantia M.O. (2020). Micromechanical investigation of grouting in soils. Int. J. Solids Struct..

[33] Nichols S.C., Goodings D.J. (2000). Physical model testing of compaction grouting in cohesionless soil. J. Geotech. Geoenviron. Eng..

[34] Guo S., Zhang Y., Wang K., Bu Y., Wang C., Ma C., Liu H. (2019). Delaying the hydration of Portland cement by sodium silicate: Setting time and retarding mechanism. Constr. Build. Mater..

[35] Liu X., Qu H., Liu B., Zhou Y., Li J., Deng W., Tao W. (2025). Research on rheological behavior and strength characteristics of cement-based grouting materials. Buildings.

[36] Zang H., Wang S., Carter J.P. (2024). Analysis of thixotropy of cement grout based on a virtual bond model. Acta Geotech..

