# Supplementary material for: Material–Medium Performance Transferability of Two Modified Cementitious Grout Systems
Source: Materials (Basel). 2026 Oct 28;19(21):4545. doi: 10.3390/ma19214545 (PMC13764444; doi:10.3390/ma19214545)
Supplement: Supplementary file 1 [file materials-19-04545-s001.zip › materials-4637308-supplementary.pdf]

Supplementary Materials: Material–Medium Performance Transferability of Two Modified Cementitious Grout Systems

Qizhi Chen [1,2], Zhipeng Shu [1,2], Hongruyi Wei [3], Zhongen Qin [1,2], Baoping Zou [1,2] and Shuyi Kong [1,2,*]

[1] School of Civil Engineering and Architecture, Zhejiang University of Science and Technology, No. 318 Liuhe Road, Xihu District, Hangzhou, 310023, China; chenqizhi@zust.edu.cn (Q.C.); 222502859020@zust.edu.cn (Z.S.)

[2] Zhejiang-Singapore Joint Laboratory for Urban Renewal and Future City, Zhejiang University of Science and Technology, Hangzhou, 310023, China

[3] Taizhou Traffic Engineering Test Center Co., Ltd., No. 168 Chunchao Road, Jiaojiang District, Taizhou, 318001, China

[*] Correspondence: kbw@zust.edu.cn

This Supplementary Information provides supporting results and calculations for material screening and confined-sand v alidation: Stage II–III central responses, Stage I stress histories, post-test grout body measurement records, the normalized screening matrix, screening-rule sensitivity, formulation mass accounting, and Stage I processing-window sensitivity. Experimental definitions and the primary interpretation are presented in the main text.

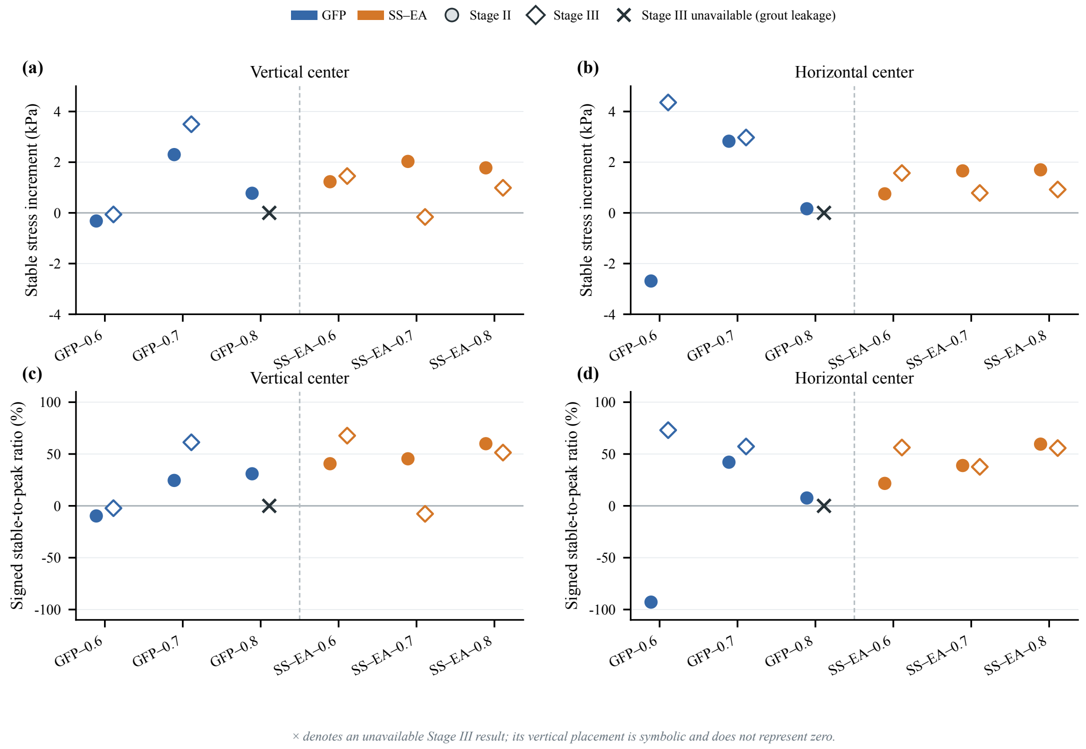

Figure S1. Stage II–III central responses for the six conditions. (a, b) Stable stress increments at vertical cell 3# and horizontal cell 8#. (c, d) Corresponding signed stable-to-peak ratios. Blue and orange denote GFP and SS-EA; solid circles and open diamonds denote Stages II and III. Gray horizontal lines indicate $y = 0$. Negative values indicate response reversal relative to the stage baseline or stress redistribution. GFP–0.8 has no valid Stage III result because of grout leakage. Dark gray crosses denote missing results only; their vertical positions do not encode a numerical value, particularly zero. Stage I selection metrics are shown in Figure 8 of the main text.

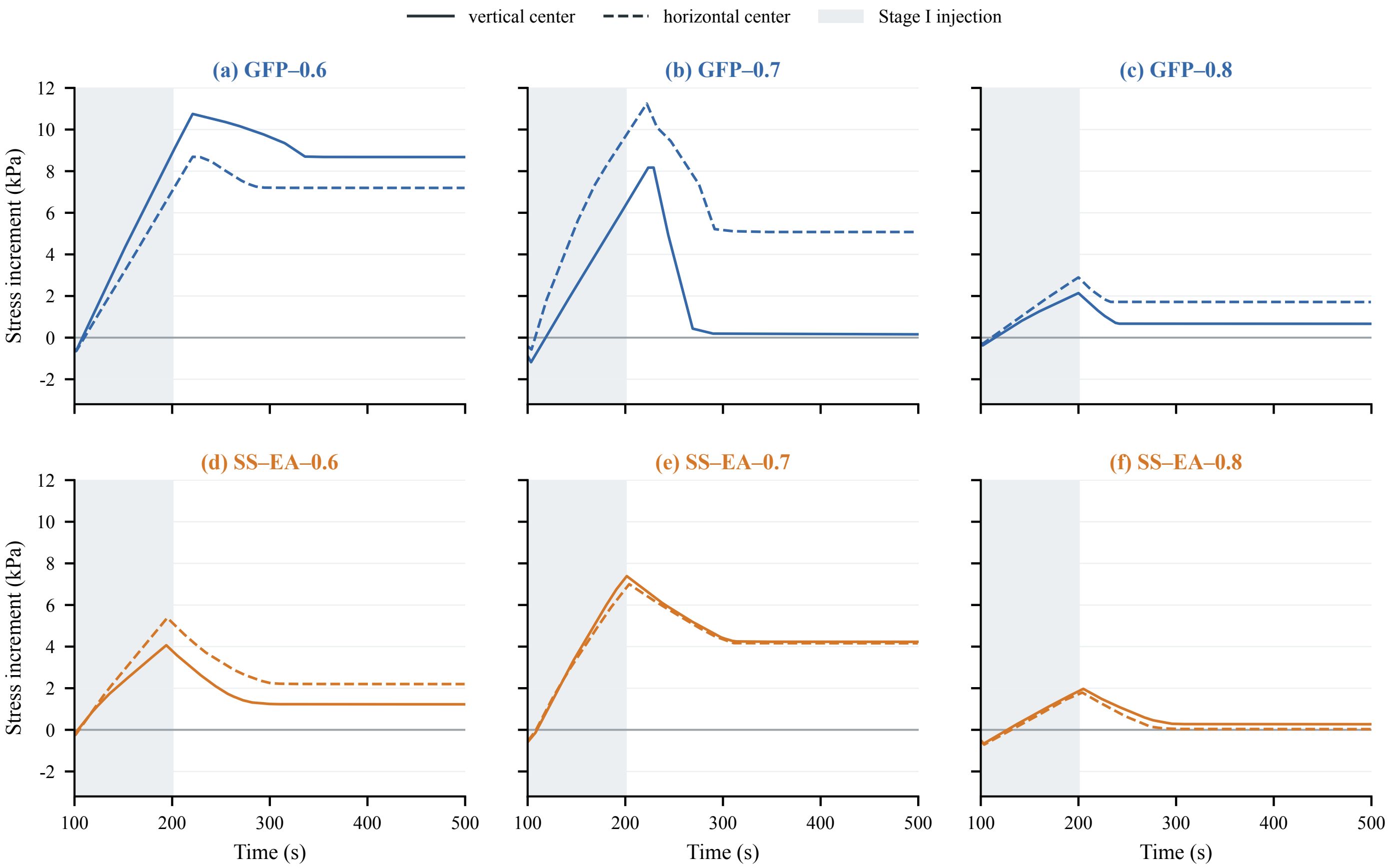

Figure S2. Stage I central stress-increment histories for the six conditions. Panels (a–f) show GFP–0.6, GFP–0.7, GFP–0.8, SS-EA–0.6, SS-EA–0.7, and SS-EA–0.8, respectively. Solid and dashed lines denote vertical cell 3# and horizontal cell 8#. Neutral gray shading marks the 1.0 L/100 s injection period. Curves show increments in the 2 Hz records relative to the mean baseline over the preceding 20 s, without smoothing or interpolation. All panels use horizontal and vertical ranges of 100–500 s and −3.2–12.0 kPa. Gray horizontal lines indicate zero increment.

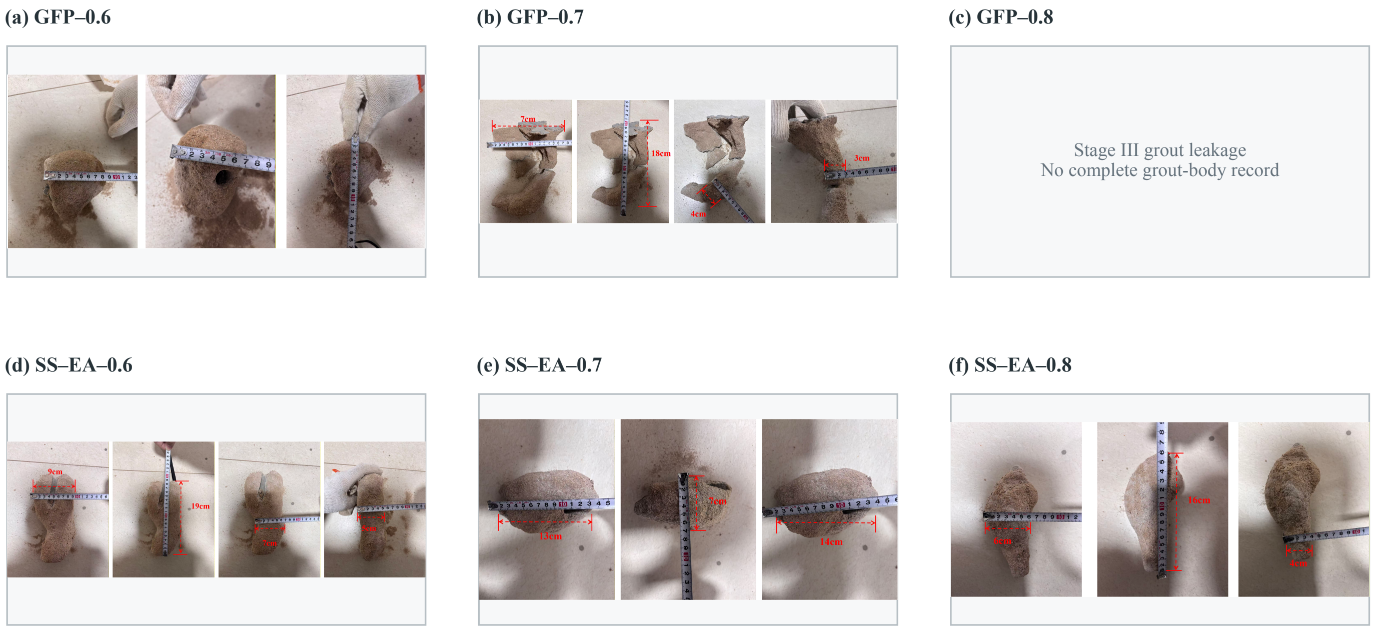

Figure S3. Post-test ruler-measurement records after the staged protocol and unloading for (a) GFP–0.6, (b) GFP–0.7, (c) GFP–0.8, (d) SS-EA–0.6, (e) SS-EA–0.7, and (f) SS-EA–0.8. For the five recovered bodies, the available viewing directions, physical rulers, and dimension annotations are shown. GFP–0.8 has no complete grout body record because of Stage III grout leakage. Representative views from these records are shown in Figure 9 of the main text.

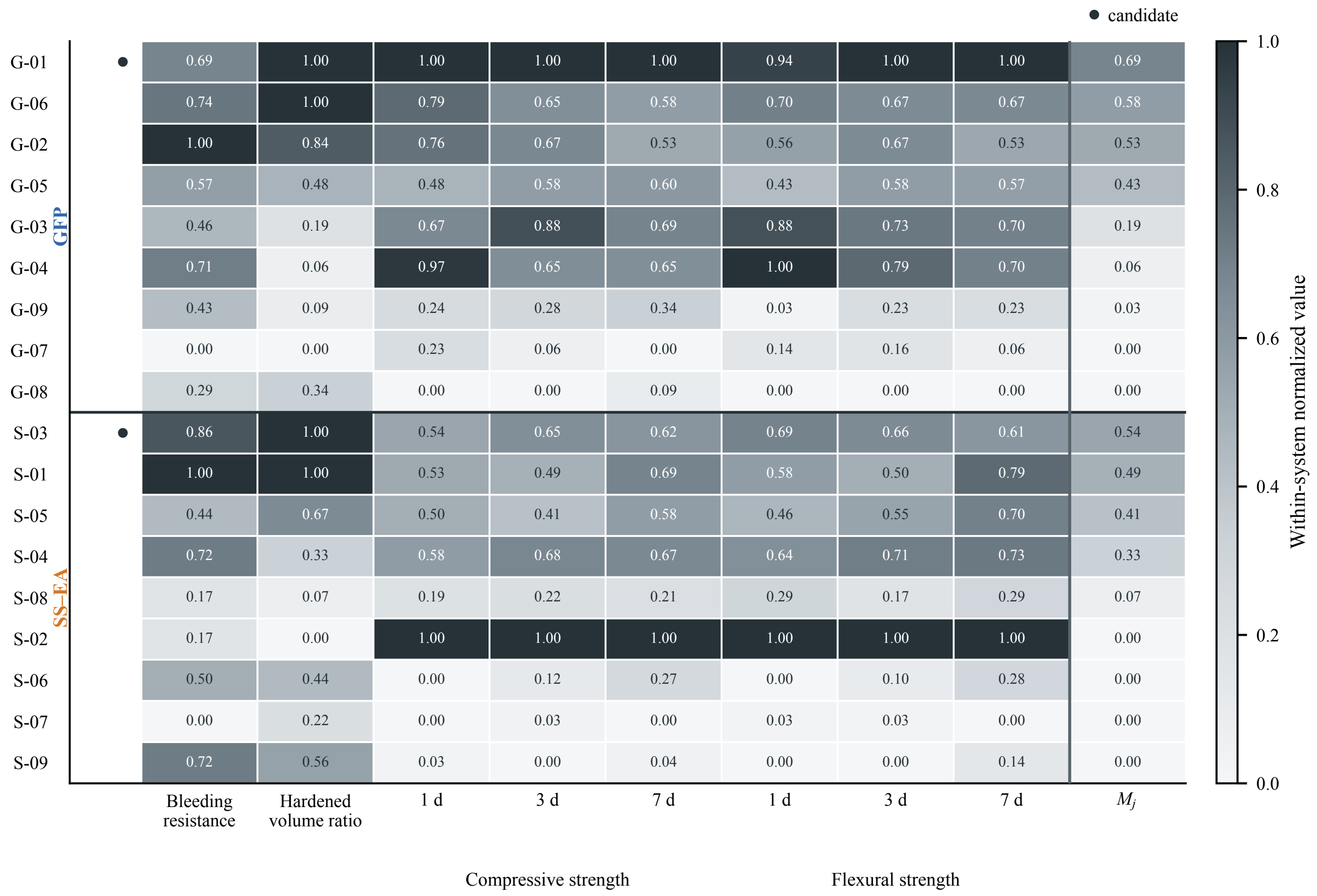

Figure S4. Complete matrix of normalized material-screening indicators and noncompensatory scores. Values were normalized to 0–1 separately within GFP and SS-EA. The final column, M_j, is the minimum of the eight normalized indicators for each mix and is separated from them by a vertical line. The grayscale range is 0–1, and the row order matches Figure 2 of the main text. Cell values are shown to two decimal places. Small solid black dots identify the two candidates, G-01 and S-03.

Screening-rule sensitivity

Screening sensitivity used the same system-specific min–max normalization as the primary analysis. The original eight-indicator maximin rule was compared with eight leave-one-indicator-out rules. Additive scores were also calculated as

$$A_j(\lambda) = \frac{\lambda}{2}(N_{j,B} + N_{j,S}) + \frac{1-\lambda}{6} \sum_{k \in \text{strength}} N_{j,k}, \qquad \lambda \in \{0.25, 0.50, 0.75\}.$$

Here, $N_{j,B}$ is normalized bleeding resistance, $N_{j,S}$ is normalized hardened volume ratio, and the six strength indicators comprise compressive and flexural strengths at 1, 3, and 7 d. At $\lambda = 0.25$, all eight indicators have equal weight. At $\lambda = 0.50$, bleeding resistance, hardened volume ratio, mean normalized compressive strength, and mean normalized flexural strength each receive one quarter of the total weight. At $\lambda = 0.75$, stability receives three quarters of the total weight. The highest score within each system defines the candidate for that rule. Scores under different rules are not on a common performance scale.

Table S1. Candidates under the primary and leave-one-indicator-out maximin rules.

Rule	GFP	Score	SS-EA	Score
All eight indicators	G-01	0.6857	S-03	0.5425
Omit bleeding	G-01	0.9355	S-03	0.5425
Omit hardened volume	G-01	0.6857	S-04	0.5824
Omit flexural 1 d	G-01	0.6857	S-03	0.5425
Omit flexural 3 d	G-01	0.6857	S-03	0.5425
Omit flexural 7 d	G-01	0.6857	S-03	0.5425
Omit compressive 1 d	G-01	0.6857	S-03	0.6098
Omit compressive 3 d	G-01	0.6857	S-03	0.5425
Omit compressive 7 d	G-01	0.6857	S-03	0.5425

G-01, S-01, S-02, and S-03 have nominal $W/B = 0.6$; S-04 has nominal $W/B = 0.7$. S-04 also has a different additive combination from S-03 and is not the SS-EA–0.7 confined-sand formulation.

Table S2. Additive screening scores for the three prespecified stability weights. Bold values identify the highest score within each system and weighting rule.

System	Mix	Nominal W/B	$\lambda = 0.25$	$\lambda = 0.50$	$\lambda = 0.75$
GFP	G-01	0.6	**0.9527**	**0.9161**	**0.8795**
GFP	G-02	0.6	0.6941	0.7690	0.8439
GFP	G-03	0.6	0.6507	0.5412	0.4318
GFP	G-04	0.7	0.6930	0.5914	0.4899
GFP	G-05	0.7	0.5366	0.5321	0.5277
GFP	G-06	0.7	0.7251	0.7738	0.8226
GFP	G-07	0.8	0.0829	0.0552	0.0276
GFP	G-08	0.8	0.0896	0.1636	0.2376
GFP	G-09	0.8	0.2330	0.2414	0.2497
SS-EA	S-01	0.6	0.6972	**0.7981**	**0.8991**
SS-EA	S-02	0.6	**0.7708**	0.5417	0.3125
SS-EA	S-03	0.6	0.7042	0.7797	0.8551
SS-EA	S-04	0.7	0.6339	0.5985	0.5631
SS-EA	S-05	0.7	0.5393	0.5447	0.5501
SS-EA	S-06	0.7	0.2133	0.2996	0.3859
SS-EA	S-07	0.8	0.0392	0.0632	0.0872
SS-EA	S-08	0.8	0.2016	0.1733	0.1450
SS-EA	S-09	0.8	0.1859	0.3369	0.4879

G-01 remains the GFP candidate for all three weights. The SS-EA candidates are S-02 at $\lambda = 0.25$ and S-01 at $\lambda = 0.50$ and 0.75. These candidates retain nominal $W/B = 0.6$ but differ in additive composition from S-03.

Formulation mass accounting

Table S3 reports component masses on normalized calculation bases of 1000 g OPC+PFA for GFP and 1000 g OPC for SS-EA. Powders are treated as dry, and SS solution contains 35.5% solids and 64.5% water. The calculated water-to-solid ratio includes SS water and solution solids on this formulation basis.

Table S3. Normalized component masses (g) and calculated water-to-solid ratios for 18 material mixes and six injection conditions.

Mix	W/B	OPC	PFA	GFP	UEA	SS	W_a	W_{SS}	S_{SS}	W_t	S_t	w/s_{calc}
G-01	0.6	900	100	5	0	0	600	0	0	600	1005	0.5970
G-02	0.6	850	150	15	0	0	600	0	0	600	1015	0.5911
G-03	0.6	800	200	10	0	0	600	0	0	600	1010	0.5941
G-04	0.7	900	100	15	0	0	700	0	0	700	1015	0.6897
G-05	0.7	850	150	5	0	0	700	0	0	700	1005	0.6965
G-06	0.7	800	200	10	0	0	700	0	0	700	1010	0.6931
G-07	0.8	900	100	10	0	0	800	0	0	800	1010	0.7921
G-08	0.8	850	150	15	0	0	800	0	0	800	1015	0.7882
G-09	0.8	800	200	5	0	0	800	0	0	800	1005	0.7960
S-01	0.6	1000	50	0	40	20	600	12.9	7.1	612.9	1097.1	0.5587
S-02	0.6	1000	25	0	30	25	600	16.125	8.875	616.125	1063.875	0.5791
S-03	0.6	1000	75	0	50	15	600	9.675	5.325	609.675	1130.325	0.5394
S-04	0.7	1000	50	0	40	20	700	12.9	7.1	712.9	1097.1	0.6498
S-05	0.7	1000	75	0	50	15	700	9.675	5.325	709.675	1130.325	0.6279
S-06	0.7	1000	25	0	30	25	700	16.125	8.875	716.125	1063.875	0.6731
S-07	0.8	1000	50	0	40	20	800	12.9	7.1	812.9	1097.1	0.7410
S-08	0.8	1000	25	0	50	15	800	9.675	5.325	809.675	1080.325	0.7495
S-09	0.8	1000	75	0	30	25	800	16.125	8.875	816.125	1113.875	0.7327
GFP–0.6	0.6	900	100	5	0	0	600	0	0	600	1005	0.5970
GFP–0.7	0.7	900	100	5	0	0	700	0	0	700	1005	0.6965
GFP–0.8	0.8	900	100	5	0	0	800	0	0	800	1005	0.7960
SS-EA–0.6	0.6	1000	75	0	50	15	600	9.675	5.325	609.675	1130.325	0.5394
SS-EA–0.7	0.7	1000	75	0	50	15	700	9.675	5.325	709.675	1130.325	0.6279
SS-EA–0.8	0.8	1000	75	0	50	15	800	9.675	5.325	809.675	1130.325	0.7163

SS denotes total solution mass. W_a is separately added water, $W_{SS} = 0.645 m_{SS}$, and $S_{SS} = 0.355 m_{SS}$. $W_t = W_a + W_{SS}$ and $S_t = m_{OPC} + m_{PFA} + m_{GFP} + m_{UEA} + S_{SS}$. Unused components are zero.

Stage I processing sensitivity

For each condition and direction, the Stage I peak used the unchanged $[100, 500)$ s interval. A common window length $L = 10, 20$, or 40 s defined the baseline $[100 - L, 100)$ and final window $[500 - L, 500)$ s. All six time records have 0.5 s spacing, with complete central-cell values in these intervals. Window means use exactly $2L$ observations, without smoothing, interpolation, or record extension.

Table S4. Stage I window sensitivity of central stress increments, retention ratios, and directional differences. The 20 s rows are the primary analysis.

Condition	L (s)	$\Delta\sigma_{s,v}$	$\Delta\sigma_{s,h}$	R_v	R_h	D_σ	D_R
GFP–0.6	10	8.957	7.452	81.209	83.281	1.504	2.072
GFP–0.6	20	8.679	7.195	80.723	82.787	1.483	2.063
GFP–0.6	40	8.113	6.679	79.654	81.701	1.434	2.047
GFP–0.7	10	0.588	5.277	6.838	46.105	4.689	39.267
GFP–0.7	20	0.158	5.077	1.936	45.146	4.919	43.210
GFP–0.7	40	-0.702	4.480	-9.596	42.070	5.182	51.666
GFP–0.8	10	0.818	1.833	35.652	60.792	1.015	25.140
GFP–0.8	20	0.665	1.713	31.059	59.170	1.048	28.112
GFP–0.8	40	0.359	1.473	19.574	55.486	1.114	35.912
SS-EA–0.6	10	1.327	2.309	31.866	42.035	0.982	10.169
SS-EA–0.6	20	1.227	2.199	30.192	40.852	0.972	10.660
SS-EA–0.6	40	1.016	1.976	26.357	38.300	0.961	11.944
SS-EA–0.7	10	4.518	4.428	58.865	60.920	0.090	2.055
SS-EA–0.7	20	4.230	4.161	57.262	59.428	0.069	2.166
SS-EA–0.7	40	3.655	3.626	53.651	56.075	0.028	2.424
SS-EA–0.8	10	0.511	0.281	23.122	13.777	0.230	9.345
SS-EA–0.8	20	0.271	0.033	13.770	1.831	0.238	11.939
SS-EA–0.8	40	-0.208	-0.466	-13.973	-36.033	0.257	22.060

Stable increments and D_σ are in kPa; R is in percent and D_R in percentage points. Negative increments and ratios are retained as baseline-relative outcomes. All four directional metrics favor GFP–0.6 and SS-EA–0.7 in their respective systems for every checked window. At 40 s, the vertical GFP–0.7 response and both SS-EA–0.8 responses change sign.
